# Influence of bone-specific material properties on finite element modelling of the skull

**DOI:** 10.1098/rsos.252468

**Published:** 2026-10-14

**Authors:** Linjie Wang, Roger Kissane, Michael J. Fagan, Graham Askew, Karl Bates, Peter Watson

**Affiliations:** ^1^ School of Mechanical Engineering, University of Leeds, Leeds, UK; ^2^ School of Biomedical Sciences, University of Leeds, Leeds, UK; ^3^ Department of Musculoskeletal & Ageing Science, University of Liverpool, Liverpool, UK; ^4^ School of Engineering, University of Hull, Hull, UK

**Keywords:** finite element analysis, material properties, bone biomechanics, skull

## Abstract

The material properties of bone in the skull are known to be heterogeneous and orthotropic, although datasets detailing these properties on a species level are limited. Therefore, computational modelling frequently uses homogeneous, isotropic material properties to analyse stresses/strains in the skull. However, it is often unknown how the choice of bone/s from which the elastic modulus is measured, or the direction in which it is measured, influences predicted stresses/strains. This study integrated imaging and measured muscle properties to create a subject-specific finite element model of a rabbit (*Oryctolagus cuniculus*) skull, to investigate the influence of modelling the elastic modulus of bone through varying levels of complexity. The results demonstrate the importance of modelling orthotropic material properties, if known. Strain distribution was insensitive to whether the same isotropic elastic modulus was used for all bones in the skull versus modelling bone-specific isotropic elastic modulus. Strain distribution was also similar when using an isotropic elastic modulus based on measurement in different orthotropic directions, albeit with a slight difference when measured in the medio-lateral direction. This highlights that isotropic, homogeneous modelling of the skull is appropriate when analysing strain distribution, although alternative material modelling should be considered when strain magnitude is of particular interest.

## 1. Introduction

Biomechanical studies of anatomical structures often use the computational technique of finite element (FE) analysis, which provides a non-invasive method of revealing stresses and strains in soft and hard tissues under physiological loading [1–8]. FE analysis is commonly used in the field of skull biomechanics, where it has been applied in a range of taxa to investigate comparative anatomy [9–12], functional anatomy in relation to feeding [13–22], physiological disorders [23–25] and medical implants [26–35].

The material properties (e.g. Young's modulus and Poisson's ratio) of soft and hard tissue are an essential input for FE studies, and the outputs of the computational modelling are directly influenced by the choice of material properties used. The material properties of bone in the skull have been reported, and like many other anatomical structures, it displays regional variation and is anisotropic [36–41]. Some FE studies of the skull have attempted to model the heterogeneous [19,42,43] and orthotropic [44,45] nature of the bone, with noticeable differences in outputs when compared with more simplified homogeneous, isotropic modelling [43–45].

Datasets of the heterogeneous and anisotropic properties of bone in the skull are sparse and limited to a small number of species. Therefore, the use of homogeneous, isotropic material properties in FE studies of the skull is often unavoidable and prevails across the literature [9–17,20–26,29,33–35,46]. Furthermore, FE studies of one species sometimes rely on using skull bone material properties from another species [14,18,20,22,46].

Nanoindentation is a popular experimental technique for measuring the elastic modulus of bone [41,47–51], and represents an accurate method of generating input data for FE modelling. Consequently, homogeneous, isotropic modelling of bone frequently defines an averaged elastic modulus from such experimental techniques. However, the averaged elastic modulus defined is dependent not only on the bone/s analysed, but also on the direction in which the modulus is measured. This is particularly important when considering the skull; for example, in the rabbit the elastic modulus in the medio-lateral direction is lower than other orthotropic directions [52], thus assigning an elastic modulus based on a single location or direction can under- or over-estimate the true stiffness of the bone in other regions.

There is no current consensus as to which bone, or direction of measurement, to assign a homogeneous, isotropic elastic modulus, on a species level, when performing FE modelling of the skull. Therefore, this study investigated the influence of modelling the elastic modulus of bone through varying levels of complexity (i.e. differing levels of regional and directional variation) upon strains in the bone. Comparison between predicted strains and those measured experimentally during biting enabled evaluation of model accuracy when using varying complexity of material modelling, thus establishing scenarios when homogeneous, isotropic modelling may be suitable when analysing skull biomechanics, and identifying the importance of considering regional/directional variation.

This study is based on modelling of a rabbit *(Oryctolagus cuniculus)* skull. The rabbit is used as a case study due to its interest in numerous scientific fields: firstly, to investigate form–function relationships and the evolution of novel morphological features, such as the biomechanical relevance of the fenestrated rostrum; secondly, understanding the healthy rabbit masticatory system is a prerequisite for several hypotheses of dental disease [53–55], one of the most commonly reported diseases in the rabbit; thirdly, to develop computational models of the rabbit that can simulate the healthy rabbit masticatory system [20,56], which have a potential application in the replacement, refinement and reduction (3Rs) of experiments in biomedical and veterinary research; and finally, the rabbit is known to possess heterogeneous and anisotropic material properties [52].

## 2. Methodology

All experimental procedures were performed in accordance with the UK Animal Scientific Procedures Act (1986) and approved by the Universities of Liverpool and Leeds Animal Welfare and Ethical Review Committees. This work conforms to the ethical requirements outlined by the journal, and is presented in accordance with the guidelines for animal work [57].

A total of six New Zealand white rabbits (Envigo; 2.679 ± 0.132 kg) were used in this study: two rabbits for *in vivo* measurement of bite force and bone strain; one sacrificed rabbit for *ex vivo* measurement of muscle morphological properties, and three sacrificed rabbits for measurement of bone properties. Rabbits used for *in vivo* measurements were housed under a 12-hour light : dark cycle at 21°C and had ad libitum access to food and water.

### 2.1. Measurement of muscle morphological properties

The skull of a rabbit was initially magnetic resonance imaging (MRI) scanned using a Siemens 3.0 T Prisma scanner (Siemens), with a human wrist coil (Hand/Wrist 16, A 3T Tim coil) at an isotropic resolution of 0.23 mm. Volumetric models of the skull, jaw and individual masticatory muscles were constructed in AVIZO 3D (v. 2021, Visualization Sciences Group Inc., USA), with muscle segmentation based on the descriptions of Weijs and Dantuma [58].

The masticatory muscles of the same individual were measured *in situ* and carefully dissected from neighbouring muscles. Muscles were weighed and then pinned out in a Sylgard-lined Petri dish at resting muscle length. Muscles were then submerged in 4% buffered paraformaldehyde for 24 hours at room temperature [59]. Fixed muscles then underwent digestion of connective tissue in a 30% nitric acid solution for 48 hours at room temperature. Digestion was arrested by suspension of fibres in a 50% glycerol solution. Muscle fibre length was determined from the average of 10–30 muscle fibres. Physiological cross-sectional area (PCSA) was calculated for each muscle using equation (2.1),

(2.1) $$\begin{array}{r} \text{PCSA}=\frac{M_{m}*\text{cos}\beta }{m_{l}*\rho } \end{array}$$

where *M_m_* is muscle mass, cos β describes the pennation angle of the muscle fibres relative to the axis of force generation (muscle fibre pennation angle was measured relative to the line of action of the muscle *in vivo)*, *m_l_* is muscle fibre length, and *ρ* is muscle density (taken as 0.001056 g mm^−^^3^ [60]). It was not possible to measure the muscle properties of the anterior zygomaticomandibularis, deep temporalis and lateral pterygoid; therefore, data from these muscles were taken from the literature. The maximum muscle force of each muscle was then calculated (table 1) by multiplying the PCSA by a muscle stress of 25 N cm^−^^2^ [61].

**Table 1.** Measured properties and calculated maximum muscle force of the rabbit masticatory muscles.

	optimal fibre length (mm)	mass (g)	optimal pennation angle (°)	maximal muscle force (N)
**superficial masseter**	13.55	2.65	18.44	43.91
**anterior deep masseter**	6.51	0.58	0	21.08
**posterior deep masseter**	8.18	0.89	0	25.75
**ant. zygo. mandibularis**	8.00^a^	0.68	–	20.12
**post. zygo. mandibularis**	6.82	0.52	0	18.04
**superficial temporalis**	7.51	0.63	0	19.85
**deep temporalis**	8.00^a^	0.94^c^	–	27.95
**medial pterygoid**	8.19	2.05	22.95	54.55
**lateral pterygoid inferior**	3.00^b^	0.41^c^	–	32.80
**lateral pterygoid superior**	3.00^b^	0.19^c^	–	15.48
**digastric**	11.57	0.77	10.72	15.47

^a^From Weijs and Dantuma [58], ^b^from Watson *et al.* [56] and ^c^estimated based on muscle volume and density of 0.001056 g mm^–3^ [60].

### 2.2. Measurement of bite forces

Two separate rabbits were anaesthetized with a subcutaneous injection of ketamine (Ketavet, Zoetis; 50 mg kg^−1^) and xylazine (Rompun, Bayer; 5 mg kg^−1^). Following confirmation of anaesthetic plane, an intravenous cannula was implanted into the posterior facial vein to maintain sedation with ketamine and xylazine throughout the experiment. Rabbits were placed into a custom-built stereotaxic frame (based on David Kopf Instruments, Model 1240 Rabbit Adaptor, Tujunga, CA, USA), securing the zygomatic arch and the skull incisors into a rigid position. Parallel platinum stimulating electrodes (0.4 mm diameter) were implanted bilaterally into the temporalis and superficial masseter muscles to stimulate across the jaw-closing musculature [62]. A custom-built bite force device made of aluminium and holding a quartz force sensor (Type 9203, Kistler), with the prongs set at 7 mm apart, was positioned between the incisors. Stimulation was delivered at 100 V for 300 ms, at 250 Hz to elicit a tetanic contraction of the whole jaw-closing musculature. The rabbit's body temperature was maintained at 37°C (Animal Temperature Controller 2000, World Precision Instruments). Following completion of the experiment, the rabbits were euthanized with an overdose of pentobarbital. Following this procedure, the maximum incisor bite force of the two rabbits was measured at 47.53 N and 57.66 N.

### 2.3. Measurement of bone strains

Bone strain was measured in the same two rabbits used during the bite force experiments. A full-length incision was performed across the skull and the skin retracted. The bone was cleared of all covering fascia, degreased using 100% ethanol on a cotton swab and left to air dry. The strain gauge rosette (three-element 45° rectangular stacked rosette, 120 Ω, Micro-Measurements, VPG) was glued onto the parietal bone using an N-butyl cyanoacrylate adhesive (Vetbond, 3M), with the centre of the gauges placed approximately 3 mm caudal of the coronal suture and 7 mm lateral of the sagittal suture. Microstrains were recorded via the System 9000 StrainSmart® Data Acquisition System (Micro-Measurements, VPG) in the StrainSmart® Data Acquisition Software, sampled at 1000 Hz.

### 2.4. Measurement of bone material properties

The elastic modulus of bones in the rabbit skull was measured using nanoindentation. A full description of the methodology is provided in Wang *et al.* [52], but is summarized here for convenience. The skulls of three further rabbits were sectioned into left and right sides, about the midline of the skull when viewed in the transverse plane. Each left/right side was set in Epo-Flo resin (MetPrep, UK), and either the anterior, medial or ventral surface of the side was polished on a Buehler Phoenix Beta grinder/polisher (Buehler, USA). A series of indentations was made on the polished surface of a side using a UNHT3 nanoindenter (Anton-Paar, Austria), using a Berkovich tip. A Berkovich tip was selected because it has been widely used previously for the indentation of bone [41,47]. Several indentations were made on the cortical bone present on the surface, along with any dentine and enamel present. The side was then ground down by approximately 1.5 mm using a Buehler Phoenix Beta grinder/polisher in a perpendicular direction to the polished surface (i.e. in either the anterio-posterior (AP), medio-lateral (ML) or ventro-dorsal (VD) direction of a global Cartesian coordinate system), and the new ground surface was then polished. Several more indentations were made on the newly polished surface; again, on the cortical bone/dentine/enamel present on the surface. This procedure was repeated throughout the side, so that each side was analysed through numerous slices. The number of indents varied per slice, as it was dependent on the area of bone/dentine/enamel present in any given slice. Hydration was maintained on each polished surface through soaking in a phosphate-buffered saline (PBS) solution for 15 minutes. The direction in which a side was measured varied between the three individuals; thus each side of the skull was analysed in three orthogonal directions: in the ML direction in all rabbits; in the AP direction in two of the rabbits; and in the VD direction in one rabbit.

A total of 2104 indentations were made on the cortical bone of 17 separate bones; while across all incisor and molar teeth, a total of 266 and 136 indentations were made on the dentine and enamel, respectively. The elastic modulus of each material was determined using the Oliver–Pharr method [63]. An average elastic modulus was subsequently calculated per bone, in each orthogonal direction (i.e. average in the AP direction, average in the ML direction and average in the VD direction). An averaged elastic modulus for enamel and dentine was calculated through averaging all moduli, irrespective of tooth location or direction of measurement. An in-built function (rmoutliers) in MATLAB (v. 2023a, The MathWorks, Inc., USA) was used to identify outliers, with the data points below the 25th percentile and above the 75th percentile of the dataset removed before calculating the average elastic modulus. After removal of outliers, the dataset consisted of 2036 indentations on the cortical bone, 257 indentations on the dentine and 132 indentations on the enamel (see table 2). The range of elastic moduli measured in these indentations is provided in the supplementary material.

**Table 2.** The total number of nanoindentations measured in each bone, dentine and enamel in each direction of measurement. The totals are the combined number of nanoindentations in three rabbits.

	number of indents		
	anterio-posterior	medio-lateral	ventro-dorsal
**alisphenoid**	19	21	5
**basisphenoid**	3	8	4
**bulla**	78	113	31
**ethmoid and internal turbinate**	16	5	18
**frontal**	113	89	67
**interparietal**	2	1	4
**maxilla**	185	173	55
**nasal**	141	52	11
**occipital**	31	61	12
**orbitosphenoid**	29	9	11
**palatine**	37	18	4
**parietal**	49	44	20
**premaxilla**	114	29	50
**presphenoid**	2	1	3
**squamosal**	59	96	56
**supraoccipital**	48	18	21
**dentine**	94	104	59
**enamel**	51	45	36

### 2.5. Multibody dynamic modelling

A subject-specific multibody dynamics analysis (MDA) model of the same individual rabbit used in the visualization and measurement of the muscle morphological properties (table 1) was created in ADAMS (v. 2021, MSC Software Corp., USA). The volumetric models of the skull and jaw were connected at the temporomandibular joint (TMJ) through a revolute joint, enabling rotation of the jaw in the sagittal plane. Muscle origin and insertion locations were determined from visual inspection of the volumetric models of each muscle. Each muscle was modelled through several individual strands, while muscle wrapping was employed to represent accurate fibre excursions and to prevent muscle–bone intersections (figure 1a). A food bolus was positioned between the upper and lower incisors and represented through two plates separated by a distance similar to the size of the force transducer used in the experimental measurement of maximum bite force. A translation spring damper was defined at coincident locations between the two plates, and set with a high stiffness (1000 N mm^−1^) to prevent relative displacement between the two plates.

**Figure 1.**
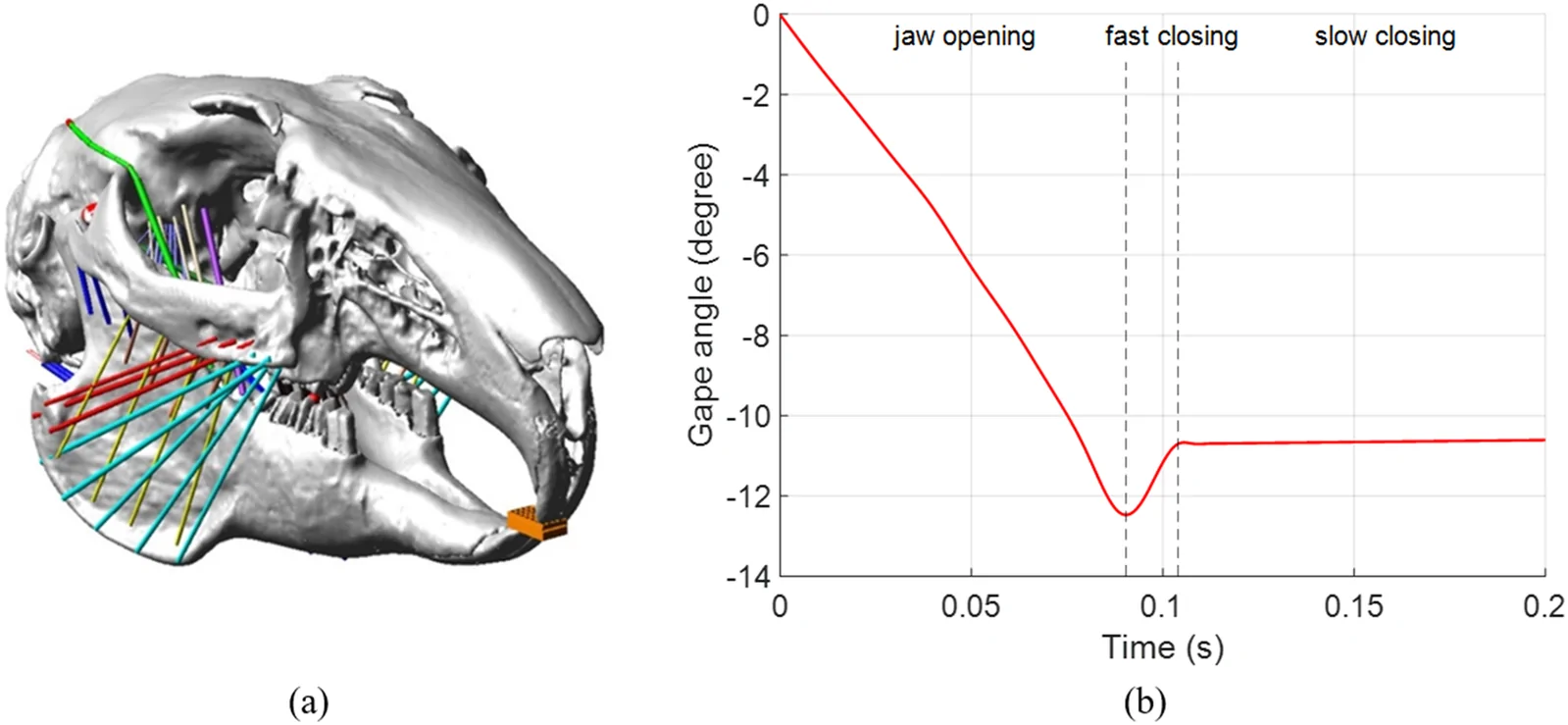
(a) The multibody dynamic musculoskeletal model of a rabbit skull and (b) motion of the jaw during an inverse dynamics simulation of a bite cycle.

An ADAMS-Simulink (Simulink, v. 2021, The MathWorks, Inc., USA) co-simulation was constructed to simulate a maximum incisor bite using a two-stage process. Stage 1: inverse dynamics initially simulated a bite cycle with three distinct phases (consistent with the description of Schwartz *et al.* [64]), (i) opening phase when the jaw opened to a gape angle of approximately 12°, (ii) fast closing phase when the jaw closed until it contacted the food bolus and (iii) slow closing phase when the jaw attempted to crush the food bolus (figure 1b). This simulation calculated the musculotendon lengths (*l_mt_*) of each muscle strand at a series of time steps during the bite cycle. The *l_mt_* values for a single muscle strand were then imported to MATLAB, where a Hill-type muscle model [65,66] in combination with the individual's optimal muscle fibre length and maximum muscle force (table 1) was used to calculate the instantaneous muscle fibre length per strand throughout the bite cycle. These instantaneous muscle fibre lengths were subsequently used to calculate muscle force-length (*ffl)* and muscle force-velocity (*ffv)* coefficients based on force-length-velocity curves (see the supplementary material, for calculation of the *ffl* and *ffv* coefficients). A maximum activation value (*a* = 1.0) was then multiplied by the *ffl* and *ffv* coefficients to calculate the maximum muscle force possible in each strand, at each time step during the slow closing phase. The muscle contractile dynamics were based on equation (2.2),

(2.2) $$F_{CE}\left(l_{m},{\overset{.}{l}}_{m},a\right)+F_{PE}\left(l_{m}\right)]*cos\beta =F_{t}\left(l_{m},l_{mt},l_{s}\right)$$

where *F_CE_* is the muscle active force (equal to *a* ∗ *ffl* ∗ *ffv*), *F_PE_* is the passive muscle force, β is the pennation angle, *F_t_* is the tendon force, *l_m_* is the muscle fibre length, $\overset{.}{\underset{m}{l}}$ is the muscle contraction velocity, and *l_s_* is the tendon slack length (see the supplementary material, for calculation of *l_s_*). Stage 2: a forward dynamics simulation was performed with the jaw originally rotated so there was a small clearance between the food bolus and the upper and lower incisors. The maximum muscle forces calculated previously in each strand were subsequently used to drive the jaw to contact the food bolus. Therefore, the forward dynamics simulation calculated the maximum bite force achievable.

### 2.6. Finite element modelling

A FE model was created through initially micro-computed tomography (µCT) scanning the same rabbit skull used to construct the MDA simulation, using a Tek HMX 160mCT scanner (X-Tek Systems Ltd, UK) with a resolution of 0.0669 mm in each direction. The µCT scan data were segmented in AVIZO 3D to identify all individual bones of the skull (figure 2 and table 3), along with sutures, periodontal ligament (PDL), enamel, dentine and tooth pulp. The resulting volumetric model was meshed with approximately 19.05 million tetrahedral elements, connected through approximately 3.76 million nodes. The mesh was imported to ANSYS (v. 2023 R1, ANSYS Inc., USA) and boundary checks confirmed there was no direct bone–bone or bone–tooth contact.

**Figure 2.**
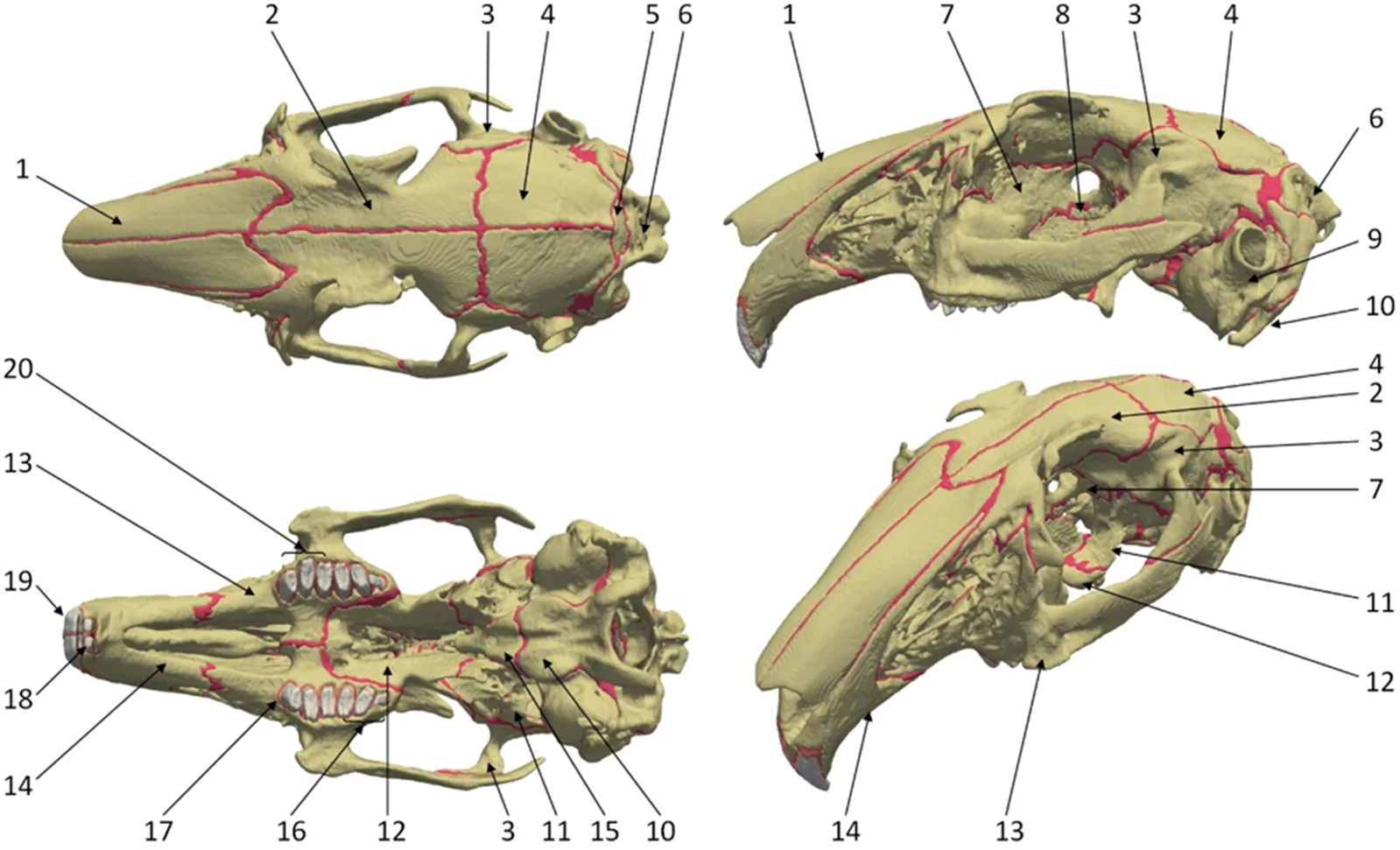
Finite element model of the rabbit showing the individual bones of the skull from which orthotropic elastic modulus properties were measured, along with the teeth, sutures and periodontal ligament. The separate materials of the skull are labelled as: 1, nasal; 2, frontal; 3, squamosal; 4, parietal; 5, interparietal; 6, supraoccipital; 7, orbitosphenoid; 8, presphenoid; 9, bulla; 10, occipital segment; 11, alisphenoid; 12, palatine; 13, maxilla; 14, premaxilla; 15, basisphenoid; 16, molars; 17, PDL; 18, incisor II; 19, incisor I; and 20, premolars.

**Table 3.** The elastic modulus (GPa) used for each material (hard and soft tissue) in nine separate homogeneous, isotropic finite element models. The average elastic modulus ($\bar{E}$) of each bone, dentine and enamel was measured using nanoindentation.

	$\bar{\text{E}}$_*Ind AP*_	$\bar{\text{E}}$_*All AP*_	$\bar{\text{E}}$_*Ind ML*_	$\bar{\text{E}}$_*All ML*_	$\bar{\text{E}}$_*Ind VD*_	$\bar{\text{E}}$_*All VD*_	$\bar{\text{E}}$_*Ind Aver*_	$\bar{\text{E}}$_*All Aver*_	*E_Datum_*
**alisphenoid**	15.79	16.85	14.51	15.79	16.05	17.26	15.22	17.19	13.7
**basisphenoid**	14.25	16.85	17.29	15.79	17.77	17.26	16.81	17.19	13.7
**bulla**	21.72	16.85	21.13	15.79	22.73	17.26	21.56	17.19	13.7
**ethmoid and internal turbinate**	16.68	16.85	13.46	15.79	16.04	17.26	15.97	17.19	13.7
**frontal**	17.13	16.85	13.08	15.79	16.83	17.26	15.72	17.19	13.7
**interparietal**	14.72	16.85	17.47	15.79	18.49	17.26	17.27	17.19	13.7
**maxilla**	19.80	16.85	17.09	15.79	17.37	17.26	18.34	17.19	13.7
**nasal**	17.36	16.85	11.14	15.79	12.24	17.26	15.49	17.19	13.7
**occipital**	15.02	16.85	15.45	15.79	17.16	17.26	15.52	17.19	13.7
**orbitosphenoid**	17.28	16.85	14.81	15.79	18.56	17.26	17.11	17.19	13.7
**palatine**	18.52	16.85	19.17	15.79	19.61	17.26	18.79	17.19	13.7
**parietal**	15.29	16.85	14.28	15.79	15.71	17.26	14.97	17.19	13.7
**premaxilla**	19.00	16.85	12.62	15.79	16.72	17.26	17.45	17.19	13.7
**presphenoid**	16.76	16.85	20.86	15.79	13.99	17.26	16.06	17.19	13.7
**squamosal**	17.22	16.85	15.94	15.79	18.52	17.26	16.99	17.19	13.7
**supraoccipital**	13.13	16.85	14.35	15.79	18.40	17.26	14.65	17.19	13.7
**vomer**	16.68	16.85	13.46	15.79	16.04	17.26	15.97	17.19	13.7
**dentine**	18.24	18.24	16.98	16.98	21.42	21.42	18.46	18.46	19.89
**enamel**	64.28	64.28	66.59	66.59	69.21	69.21	66.41	66.41	65
**PDL^a^**	0.05	0.05	0.05	0.05	0.05	0.05	0.05	0.05	0.05
**pulp^b^**	0.002	0.002	0.002	0.002	0.002	0.002	0.002	0.002	0.002
**suture^c^**	0.01	0.01	0.01	0.01	0.01	0.01	0.01	0.01	0.02

The elastic modulus of the pulp, enamel and sutures were taken from literature (^a^taken from Rees and Jacobsen [67], ^b^taken from Benazzi *et al.* [68], ^c^taken from Moazen *et al.* [69]). The elastic modulus for the vomer bone was not measured in this study and was assigned the same properties as the ethmoid and internal turbinate bone. The labelling of each model refers to: ind = the average elastic modulus defined is bone-specific; all = the same average elastic modulus defined for all bones; AP = the average elastic modulus measured in the antero-posterior direction; ML = the average elastic modulus measured in the medio-lateral direction; VD = the average elastic modulus measured in the ventro-dorsal direction.

To facilitate a comparison between predicted strains in the FE model and those measured experimentally, the bite force simulated in the FE model should be representative of the bite forces recorded during the strain measurements (since a different rabbit was used for the maximum bite force measurement). The calculated maximum bite force from the MDA model (61.85 N) was higher than the experimentally measured average maximum bite force (52.60 N). Therefore, the muscle forces calculated from the MDA model were scaled by a factor of 0.85. The scaled muscle forces calculated from the MDA simulation were applied to the FE model as a nodal load at the origin of each muscle strand. A node on the left TMJ was constrained in the AP and VD directions, while a node on the right TMJ was constrained in all degrees of freedom. Finally, a node at the tip of each incisor was constrained in the VD direction.

To test the sensitivity of the FE predictions to material properties, eight separate homogeneous, isotropic simulations were performed with the following used to define the elastic modulus for bone, dentine and enamel (table 3); ${\bar{E}}_{IndAP}$ = each material defined with its average elastic modulus ($\bar{E}$) when measured in the AP direction; ${\bar{E}}_{AllAP}$ = all bones defined with the same $\bar{E}$, calculated as the average across all bones when measured in the AP direction (16.85 GPa). Dentine and enamel are defined with their $\bar{E}$ when measured in the AP direction (18.24 and 64.28 GPa, respectively); ${\bar{E}}_{IndML}$ = each material defined with its $\bar{E}$ when measured in the ML direction; ${\bar{E}}_{AllML}$ = all bones defined with the same $\bar{E}$, calculated as the average across all bones when measured in the ML direction (15.79 GPa). Dentine and enamel defined with their $\bar{E}$ when measured in the ML direction (16.98 and 66.59 GPa, respectively); ${\bar{E}}_{IndVD}$ = each material defined with its $\bar{E}$ when measured in the VD direction; ${\bar{E}}_{AllVD}$ = all bones defined with the same $\bar{E}$, calculated as the average across all bones when measured in the VD direction (17.26 GPa). Dentine and enamel are defined with their $\bar{E}$ when measured in the VD direction (21.42 and 69.21 GPa, respectively); ${\bar{E}}_{IndAver}$ = each material defined with its $\bar{E}$ from all three orthotropic directions; ${\bar{E}}_{AllAver}$ = bone defined with the same $\bar{E}$, calculated as the average across all bones and orthotropic directions (17.19 GPa). Dentine and enamel were defined with their $\bar{E}$ across all orthotropic directions (18.46 and 66.41 GPa, respectively). All bones in all homogeneous, isotropic simulations were modelled with a Poisson ratio (ν) of 0.3.

Homogeneous, orthotropic simulations were also constructed using bone-specific material properties whereby *E_x_* corresponded to the $\bar{E}$ when measured in the AP direction, *E_y_* corresponded to $\bar{E}$ when measured in the VD direction, and *E_z_* corresponded to $\bar{E}$ when measured in the ML direction. Orthotropic shear moduli for each bone (*G_xy_*, *G_yz_* and *G_zx_*) were defined as a scaled value of the respective orthotropic values. A total of four separate homogeneous, orthotropic simulations were performed using different shear moduli scaling values: ${\bar{E}}_{IndOrth20}$ = shear moduli of each bone defined as 20 times lower than their respective orthotropic values [70]; ${\bar{E}}_{IndOrth5.5}$ = shear moduli of each bone defined as 5.5 times lower than their respective orthotropic values [71,72]; ${\bar{E}}_{IndOrth4}$ = shear moduli of each bone defined as four times lower than their respective orthotropic values [73] and ${\bar{E}}_{IndOrth3}$ = shear moduli of each bone defined as three times lower than their respective orthotropic values [74]. The resulting shear modulus applied to each bone in the homogeneous, orthotropic simulations is provided in table 4; these simulations were performed with ν = 0.3. The rabbit skull was positioned in the same orientation (with respect to a Cartesian coordinate system) as that used when measuring the elastic modulus in each orthotropic direction.

**Table 4.** The shear modulus values applied in each orthotropic direction for the separate orthotropic simulations; *x*‑axis corresponds to the antero-posterior direction, *y*‑axis corresponds to the ventro-dorsal direction, and the *z*‑axis corresponds to the medio-lateral direction.

	$\bar{E}$_*Ind Orth 20*_			$\bar{E}$_*Ind Orth 5.5*_			$\bar{E}$_*Ind Orth 4*_			$\bar{E}$_*Ind Orth 3*_		
	*G_xy_*	*G_yz_*	*G_zx_*	*G_xy_*	*G_yz_*	*G_zx_*	*G_xy_*	*G_yz_*	*G_zx_*	*G_xy_*	*G_yz_*	*G_zx_*
**alisphenoid**	0.79	0.80	0.73	2.87	2.92	2.64	3.95	4.01	3.63	5.26	5.35	4.84
**basisphenoid**	0.71	0.89	0.86	2.59	3.23	3.14	3.56	4.44	4.32	4.75	5.92	5.76
**bulla**	1.09	1.14	1.06	3.95	4.13	3.84	5.43	5.68	5.28	7.24	7.58	7.04
**ethmoid and internal turbinate**	0.83	0.80	0.67	3.03	2.92	2.45	4.17	4.01	3.37	5.56	5.35	4.49
**frontal**	0.86	0.84	0.65	3.11	3.06	2.38	4.28	4.21	3.27	5.71	5.61	4.36
**interparietal**	0.74	0.92	0.87	2.68	3.36	3.18	3.68	4.62	4.37	4.91	6.16	5.82
**maxilla**	0.99	0.87	0.85	3.60	3.16	3.11	4.95	4.34	4.27	6.60	5.79	5.70
**nasal**	0.87	0.61	0.56	3.16	2.23	2.03	4.34	3.06	2.79	5.79	4.08	3.71
**occipital**	0.75	0.86	0.77	2.73	3.12	2.81	3.76	4.29	3.86	5.01	5.72	5.15
**orbitosphenoid**	0.86	0.93	0.74	3.14	3.37	2.69	4.32	4.64	3.70	5.76	6.19	4.94
**palatine**	0.93	0.98	0.96	3.37	3.57	3.49	4.63	4.90	4.79	6.17	6.54	6.39
**parietal**	0.76	0.79	0.71	2.78	2.86	2.60	3.82	3.93	3.57	5.10	5.24	4.76
**premaxilla**	0.95	0.84	0.63	3.45	3.04	2.29	4.75	4.18	3.16	6.33	5.57	4.21
**presphenoid**	0.84	0.70	1.04	3.05	2.54	3.79	4.19	3.50	5.22	5.59	4.66	6.95
**squamosal**	0.86	0.93	0.80	3.13	3.37	2.90	4.31	4.63	3.99	5.74	6.17	5.31
**supraoccipital**	0.66	0.92	0.72	2.39	3.35	2.61	3.28	4.60	3.59	4.38	6.13	4.78
**vomer**	0.83	0.80	0.67	3.03	2.92	2.45	4.17	4.01	3.37	5.56	5.35	4.49

All simulations were assigned the same material properties for the PDL (*E* = 0.05 GPa, ν = 0.4) [67], pulp (*E* = 0.002 GPa, ν = 0.45) [68] and sutures (*E* = 0.01 GPa, ν = 0.4) [69] (table 3). The results from all simulations were compared with a datum model (*E_Datum_*) which simulated the following homogeneous, isotropic properties to match that of previous FE modelling of the rabbit skull [20]: all bone with *E* = 13.7 GPa (ν = 0.33); dentine with *E*  =  19.89 GPa (ν  =  0.31); enamel with *E*  =  65.00 GPa (ν  =  0.33); PDL with *E*  =  0.05 GPa (ν  =  0.49); pulp with *E*  =  0.002 GPa (ν  =  0.49) and sutures with *E*  =  0.02 GPa (ν  =  0.49; table 3). It should be noted that the *E_Datum_* simulation used an elastic modulus of bone that was not species-specific and comes from measurements of canine mandibular cortical bone [75].

The FE simulations in this study were designed to represent a range of potential scenarios often experienced when investigating the skull through FE analysis. The *E_Datum_* simulation mimics a common situation in zoological and comparative anatomy where species-specific material properties are often not available [14,18,20,22,46]. The isotropic simulations (e.g. ${\bar{E}}_{IndAP}$ and ${\bar{E}}_{AllAP}$) represent a situation when it is unknown if species-specific material properties should be measured from a specific bone, or even in a certain orthotropic direction, but they neglect the true orthotropic nature of bone. This is expected to generate more accurate results than modelling non-species-specific material properties. The orthotropic simulations (e.g. ${\bar{E}}_{IndOrth20}$ and ${\bar{E}}_{IndOrth5.5}$) represent the most physiological representation of the bone and are expected to generate the most accurate results of all the simulations.

To facilitate comparison between each simulation and that of *E_Datum_*, difference contour plots were created through subtracting the von Mises strain in each element of the *E_Datum_* simulation from that generated in another simulation. To further investigate differences between simulations, nodal von Mises strains were measured at various points along four paths that crossed several bones on the right side of the skull: Path 1—originating from the anterior limit of the zygomatic arch and travelling posteriorly along the zygomatic arch to the squamosal bone, terminating at the TMJ; Path 2—originating from the posterior portion of the parietal bone and moving anteriorly through the frontal bone, terminating at the anterio-medial side of the nasal bone; Path 3—originating from the posterior region of the frontal bone, travelling ventrally through the squamosal and alisphenoid bones, then moving anteriorly to terminate at the palatine bone; and Path 4—originating in the frontal bone and moving ventrally through the anterior region of the maxilla, and then posteriorly to the palatine. The average strain at each point on a path was calculated by averaging the strains recorded at the surface nodes that were associated with the elements attached to that point. This inter-model comparison considered von Mises strain because it is a popular measure used in analysis of skull biomechanics. In addition, von Mises strain is convenient because it is a scalar function combining the three principal strains, is related to the von Mises failure criterion and is useful for comparing the performance of complex three-dimensional geometries.

To assess the accuracy of the separate simulations, the average nodal principal tensile strain was recorded from an area on the left parietal bone which approximately matched that of the placement of the strain gauge in the experimental recordings.

## 3. Results

The *E_Datum_* simulation, which includes bone material properties from the canine mandible [75], produced a strain distribution with the highest strain magnitudes observed in the orbitosphenoid bone, and in concentrations travelling from the TMJ in various directions: laterally through the squamosal bone and zygomatic arch; medially through the alisphenoid and basioccipital bones; and posteriorly through the squamosal bone (figure 3).

**Figure 3.**
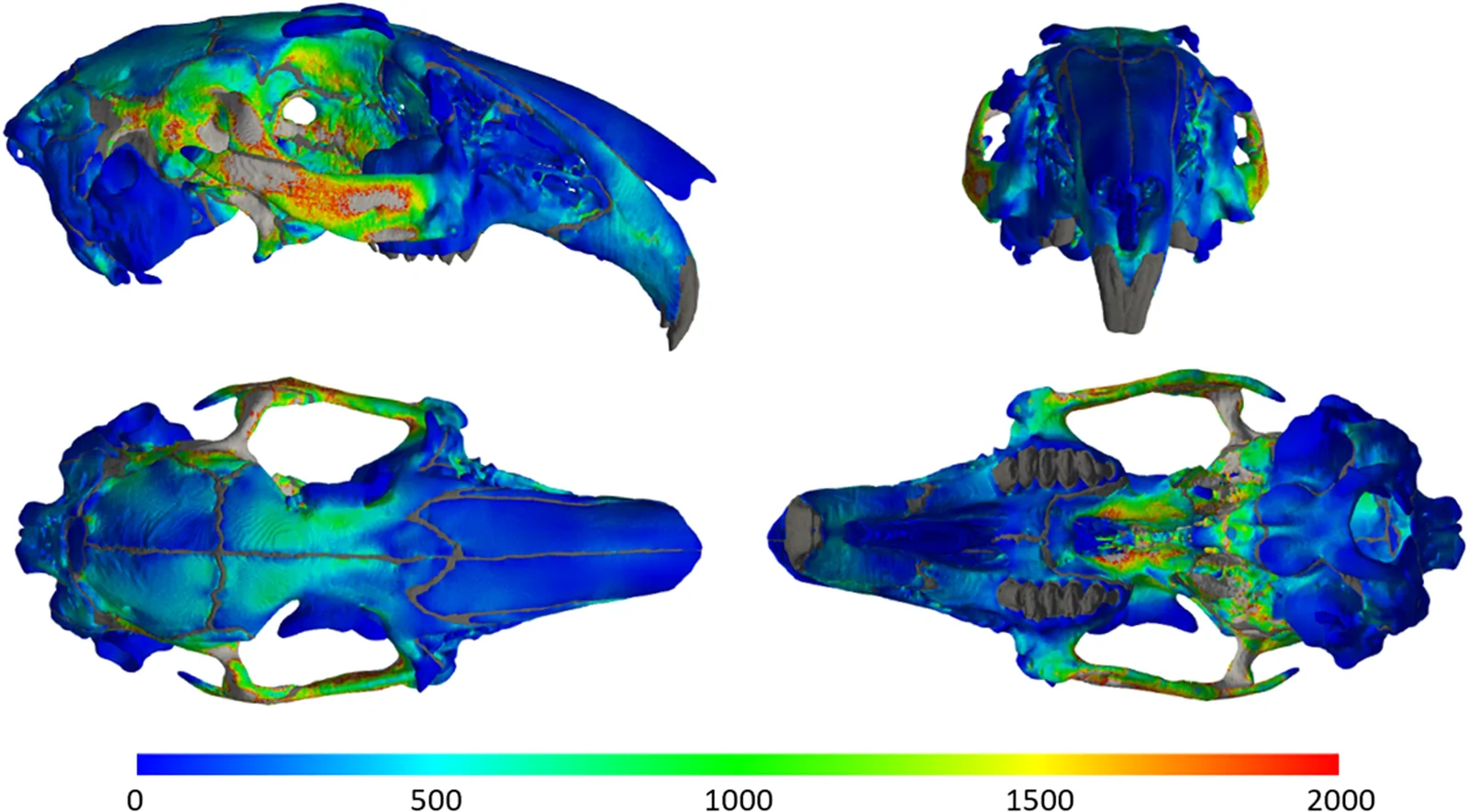
The element von Mises strain (µε) distribution generated in the datum simulation (*E_Datum_*) with a maximum incisor bite. Regions in light grey represent strains above 2000 µɛ. The teeth and sutures are displayed in dark grey.

All isotropic simulations using species-specific (rabbit) material properties (i.e. ${\bar{E}}_{IndAP}$, ${\bar{E}}_{AllAP}$, etc.) produced similar region-to-region differences in strain to those observed in the *E_Datum_* simulation (figure 4). Regions that experienced an increase/decrease in strain relative to the *E_Datum_* simulation were sporadically located throughout the skull; lower strains were observed in the posterior regions of the zygomatic arch and anterior regions of the orbitosphenoid; higher strains in the squamosal (in the posterior orbit), anterior regions of the frontal bone, premaxilla and posterior regions of the alisphenoid bone.

**Figure 4.**
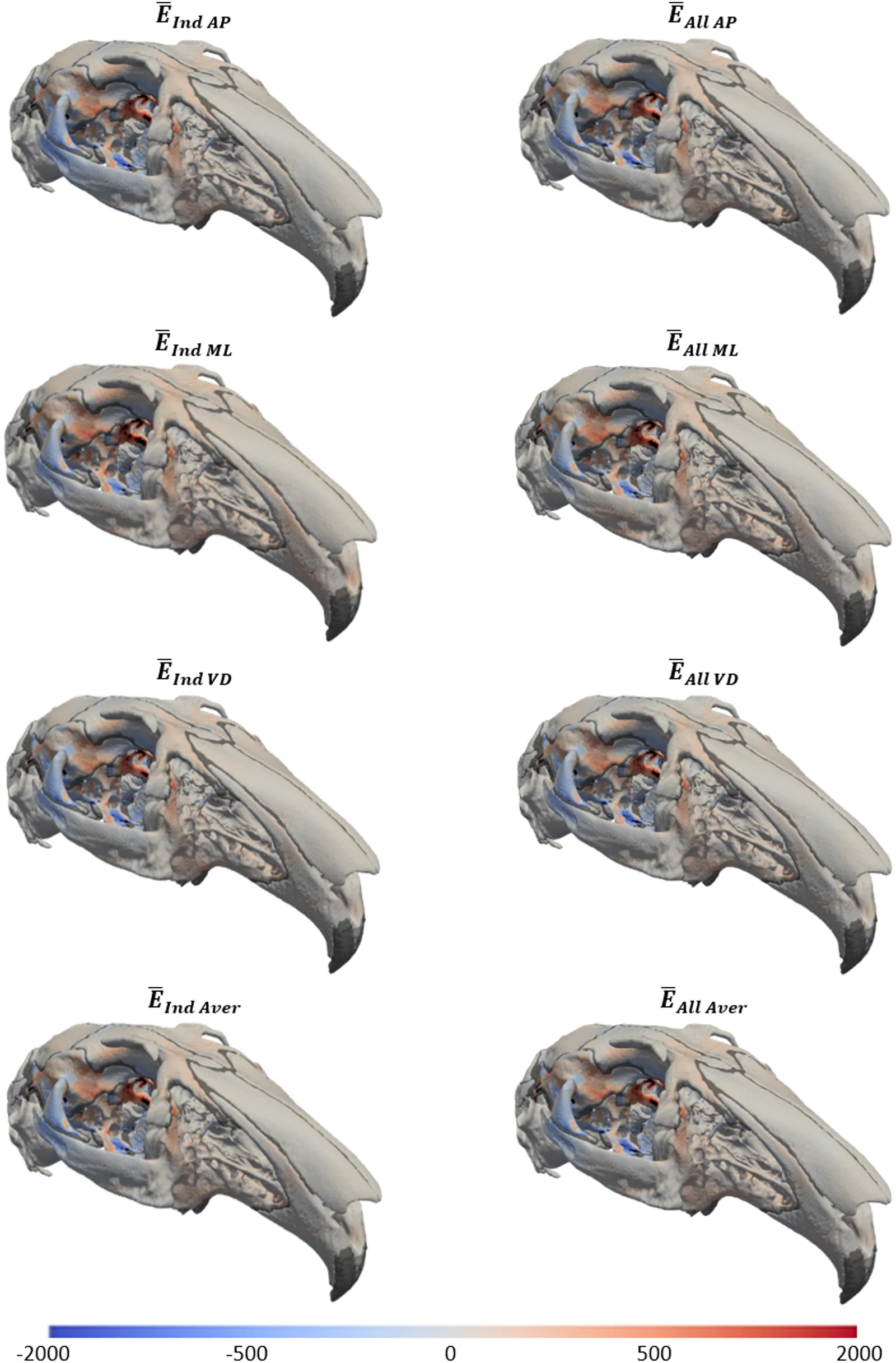
The difference in element von Mises strain between the homogeneous, isotropic simulations and that of the datum simulation (*E_Datum_*). Positive values represent where a simulation displayed strains that are larger when compared with the datum model. Negative values represent where strains are lower when compared with the datum simulation. Regions in black represent strain values exceeding ± 2000 µɛ. The teeth and sutures are shown in dark grey.

The similarity between the species-specific isotropic simulations was also illustrated in the distribution of strains along Paths 3 and 4 (figure 5). A larger deviation between these simulations was observed in Paths 1 and 2, with the simulations using properties measured in the ML direction (i.e. ${\bar{E}}_{IndML}$ and ${\bar{E}}_{AllML}$) producing higher strains, while the ${\bar{E}}_{IndAP}$ simulation generally produced lower strains. However, the deviation between the species-specific isotropic simulations and the *E_Datum_* simulation was much larger, with the latter generally producing noticeably larger strains in the posterior regions of the maxilla along Path 1 and lower strains along Paths 3 and 4 (figure 5).

**Figure 5.**
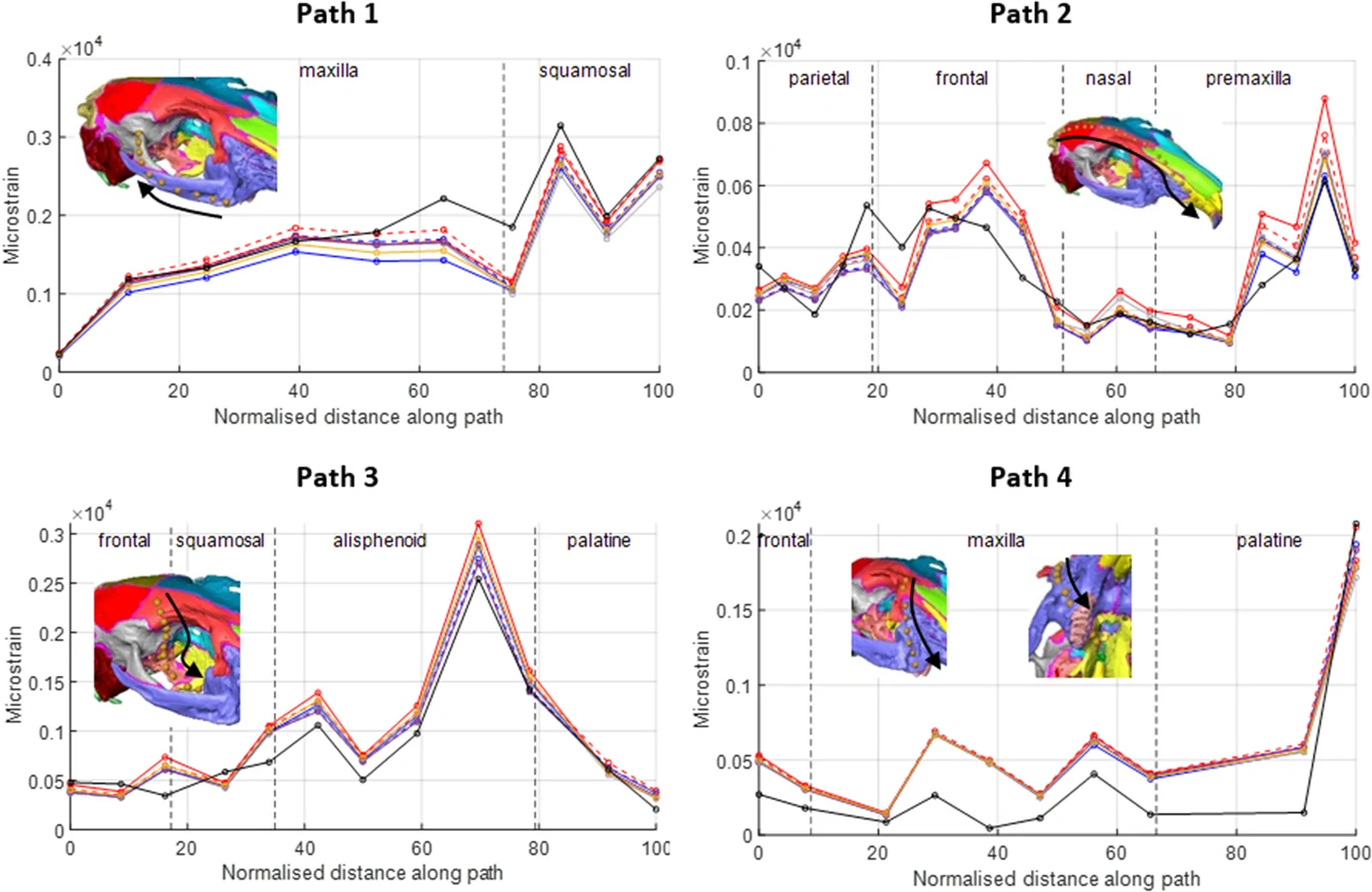
The average nodal von Mises strain at points along paths in the skull in various homogeneous, isotropic simulations. Vertical black dashed lines represent the movement of the path into another bone. Inserts illustrate the location and direction of each path, along with the points at which the average nodal strains were recorded (shown as yellow spheres). Solid dark blue line, $\bar{E}$_*Ind AP*_; dashed dark blue line, $\bar{E}$_*All AP*_; solid red line, $\bar{E}$_*Ind ML*_; dashed red line, $\bar{E}$_*All ML*_; solid green line, $\bar{E}$_*Ind VD*_; dashed grey line, $\bar{E}$_*All VD*_; solid yellow line, $\bar{E}$_*Ind Aver*_; solid purple line, $\bar{E}$_*All Aver*_; and black line, *E_Datum_*. Note—the species-specific material properties simulations produce similar strain magnitudes and often overlap.

In comparison, there was a much larger deviation in strains between the species-specific orthotropic simulations (i.e. ${\bar{E}}_{IndOrth20}$, ${\bar{E}}_{IndOrth20}$, etc.) and the *E_Datum_* simulation (figure 6). This was particularly evident in the ${\bar{E}}_{IndOrth20}$ simulation, where several regions of the skull experienced a strain difference that exceeded + 2000 µε relative to the *E_Datum_* simulation. In comparison, the other species-specific orthotropic simulations (i.e. those with a higher shear modulus) produced differences in strain that were much lower in magnitude, although they all displayed a similar distribution of the differences in relative strain. These observations were once again confirmed through analysing the strain distribution along each path (figure 7).

**Figure 6.**
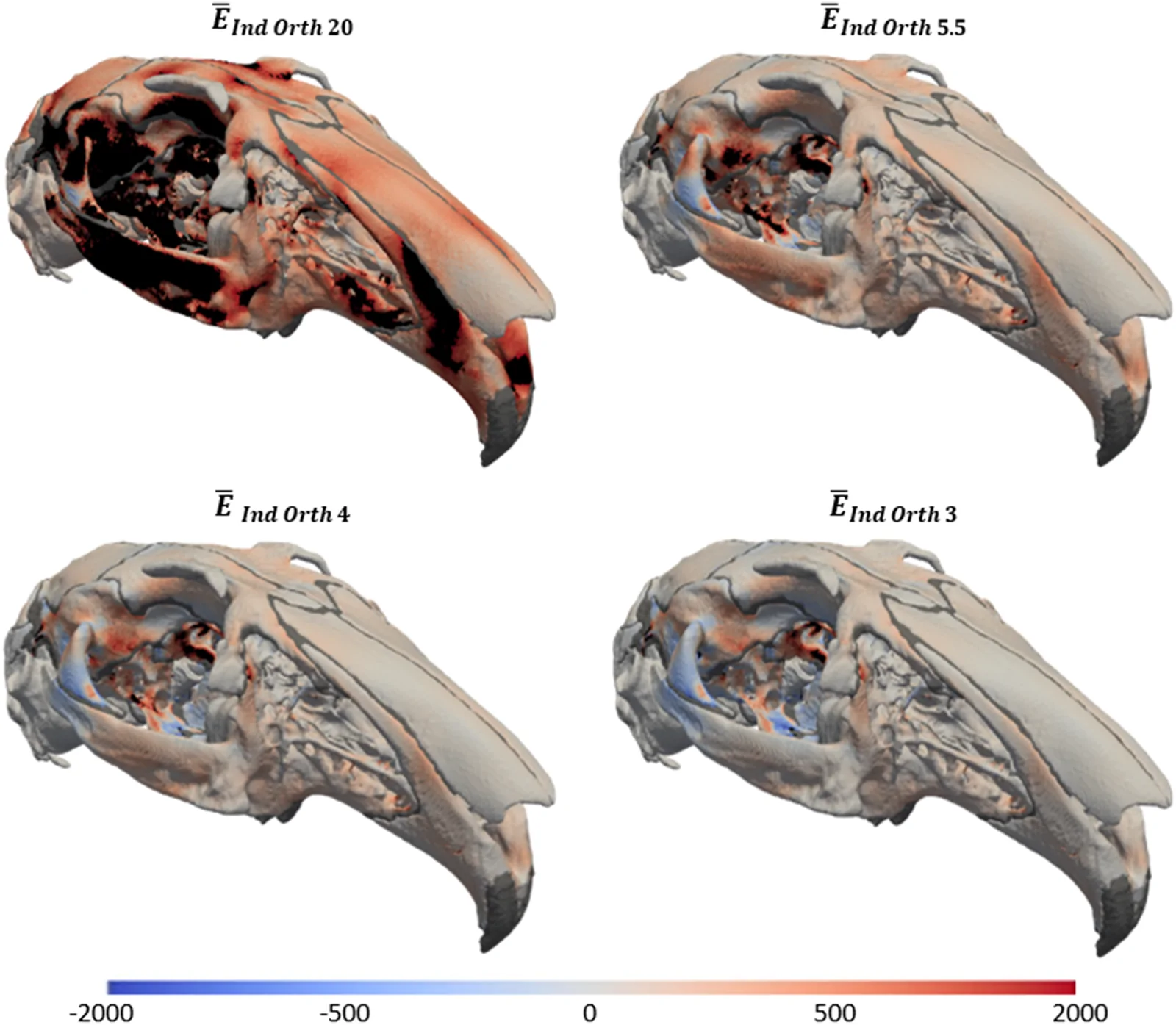
The difference in element von Mises strain between the various homogeneous, orthotropic simulations and that of the datum simulation (*E_Datum_*). Positive values represent where a simulation displayed strains that are larger when compared with the datum model. Negative values represent where strains are lower when compared with the datum simulation. Regions in black represent strain values exceeding ± 2000 µɛ. The teeth and sutures are shown in dark grey.

**Figure 7.**
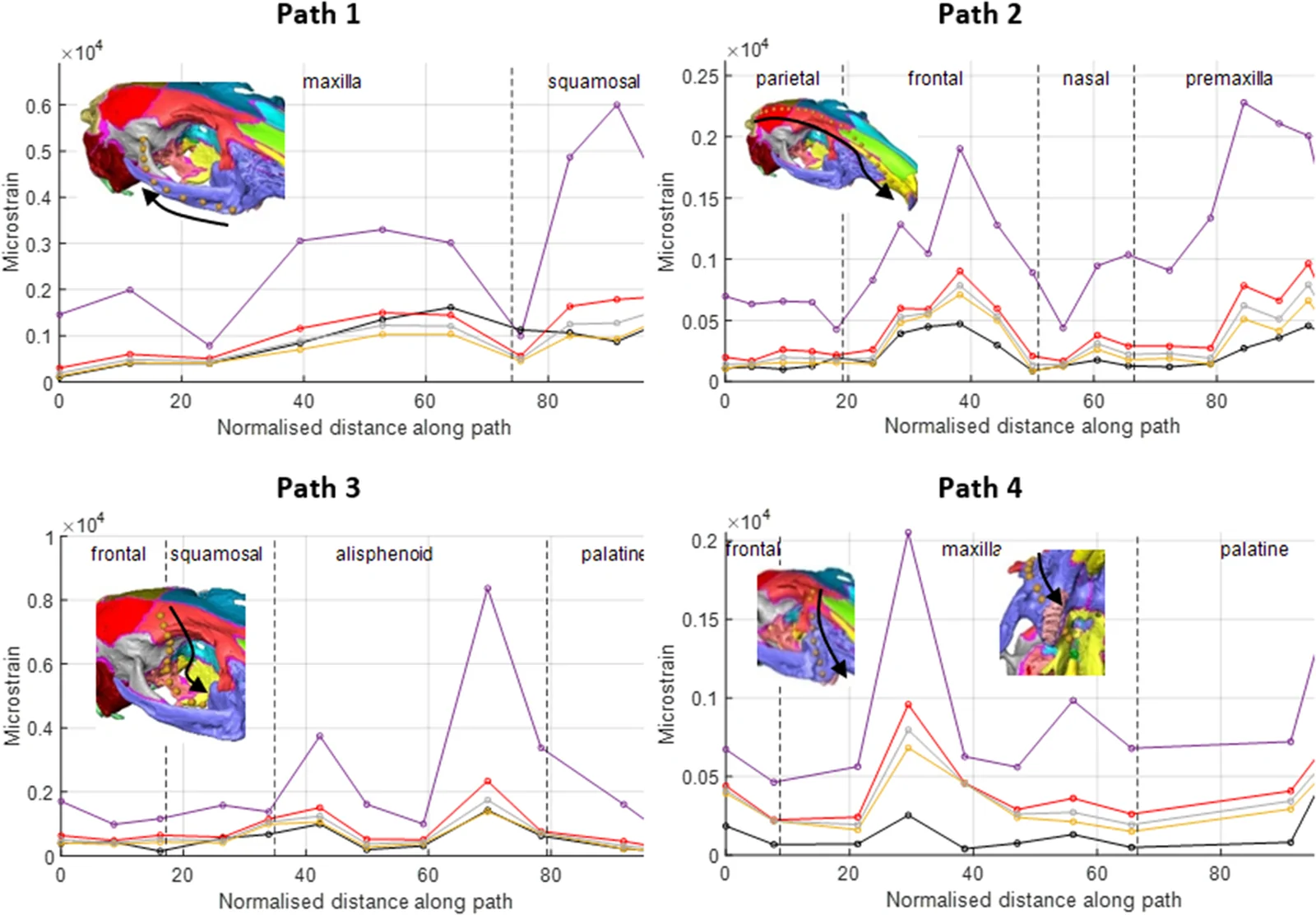
The average nodal von Mises strain at points along paths in the skull in various homogeneous, orthotropic simulations, along with the homogeneous, isotropic simulation of *E_Datum_*. Vertical black dashed lines represent the movement of the path into another bone. Inserts illustrate the location and direction of each path, along with the points at which the average nodal strains were recorded (shown as yellow spheres). Solid purple line, $\bar{E}$_*Ind Orth 20*_; solid red line, $\bar{E}$_*Ind Orth 5.5*_; solid grey line, $\bar{E}$_*Ind Orth 4*_; solid yellow line, $\bar{E}$_*Ind Orth 3*_; and black line, *E_Datum_*. Note—the species-specific material properties simulations produce similar strain magnitudes and often overlap.

The species-specific isotropic simulations recorded an average principal tensile strain in the left parietal bone ranging between 151.98 and 173.30 µε, which was lower than that measured experimentally, 316.82 µε (s.d. = 30.32; figure 8). The species-specific orthotropic simulations recorded the largest average principal tensile strain (between 177.97 and 336.83 µε), while the *E_Datum_* simulation recorded the lowest average principal tensile strain (120.39 µε; s.d. = 38.09). The ${\bar{E}}_{IndOrth20}$ simulation produced the closest average principal tensile strain to that measured experimentally, differing by only 6.32% (an increase of 20.01 µε).

**Figure 8.**
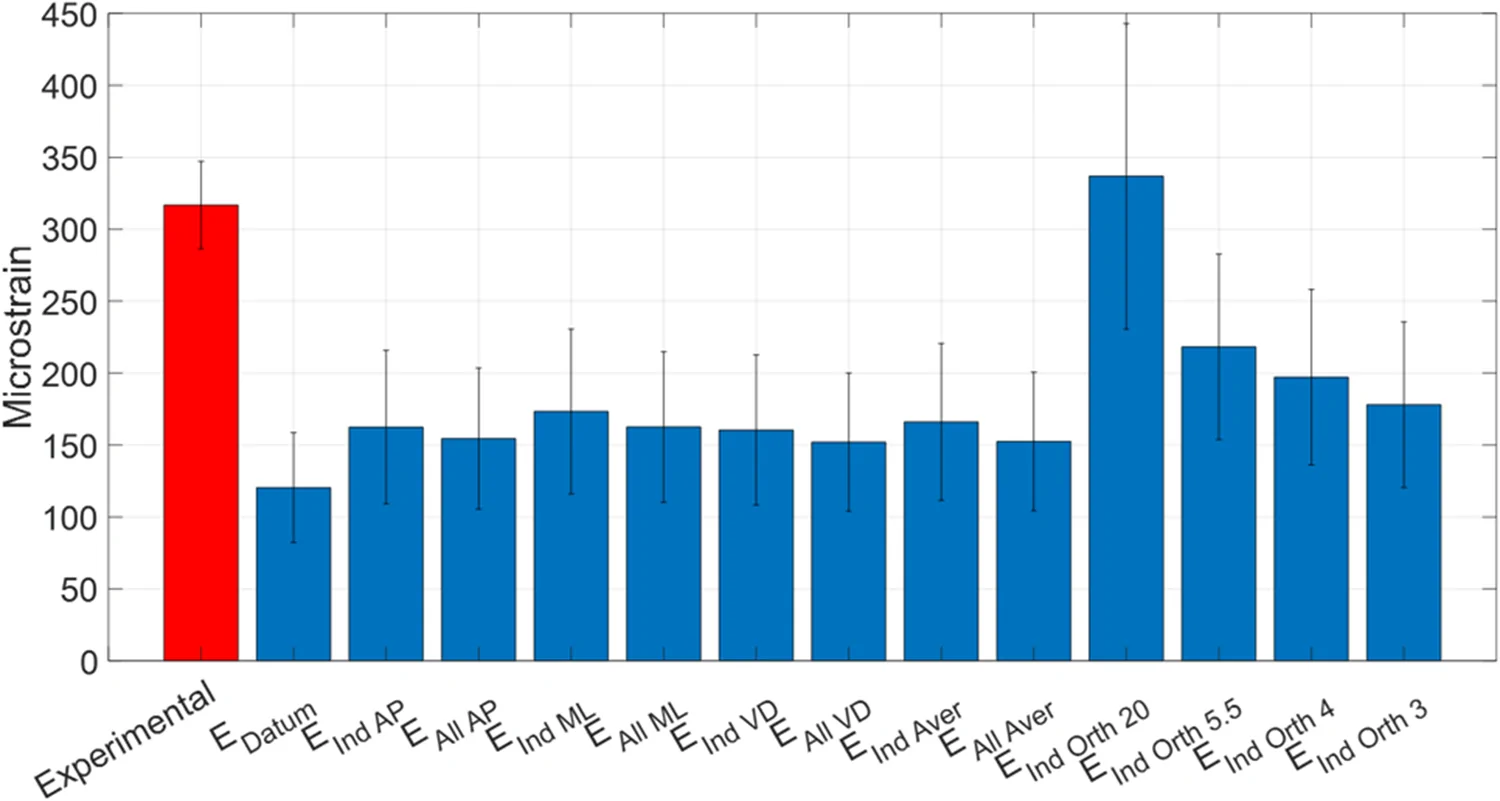
The average principal tensile strain measured experimentally through a strain gauge positioned on the left parietal bone (red bar), and the average nodal principal tensile strain recorded from a comparative location of the strain gauge in the separate FE simulations (blue bars). Error bars indicate ± 1 s.d. of the average principal tensile strain measured experimentally during a maximum incisor bite in two individuals. Error bars indicate ± 1 s.d. of the average principal tensile strain measured across the surface area of the gauge in the FE simulations.

## 4. Discussion

This study investigated the influence of material properties on skull biomechanics. Comparisons between simulations of different material properties were based on bone strains associated with mastication. The biomechanical loading used in the FE modelling was deemed appropriate, as the maximum incisor bite force used in the FE simulations was scaled to match the average force measured experimentally, and the strain distribution in the *E_Datum_* simulation (figure 3) is similar to that observed in another study modelling maximal incisor biting in the rabbit [20].

The nature of mechanical testing of material properties often means that measuring properties for the individual (or even species) under investigation is not possible, for reasons such as lack of specimen availability or feasibility to perform destructive testing. When faced with such problems, this study suggests that when performing homogeneous, isotropic simulations, the use of non-species-specific bone material properties (in this case from the canine mandible) may be suitable when studies are solely interested in general qualitative spatial variation in strain or strain distributions. This is demonstrated by the fairly consistent distribution of region-to-region differences in strain between the *E_Datum_* simulation and the species-specific isotropic simulations (figure 4). However, this observation is limited to overall strain distributions and cannot be expanded to strain magnitude as: (i) species-specific isotropic simulations often produced strain differences in excess of ± 500 µɛ when compared with the *E_Datum_* simulation—the magnitude of these differences was found to be dependent on both location in the skull and the position within a single bone itself (figure 5); and (ii) the *E_Datum_* simulation recorded an average principal tensile strain in the left parietal bone which was 61.20% lower than measured experimentally (a reduction of 196.43 µε; figure 8).

This finding that the use of non-species-specific material properties still produces comparative strain distributions is highly encouraging for comparative zoological and evolutionary studies, which often seek to understand either the relative or qualitative impact of morphological changes to skull shape on stress–strain patterns across taxa [16,76,77].

Simulating species-specific isotropic material properties was observed to produce lower strains when compared with orthotropic modelling. However, the consistent profile of the strains across the four different paths in figures 5 and 7, along with similarities between the ${\bar{E}}_{IndOrth3}$ (figure 6) and the isotropic difference plots (figure 4), suggest that isotropic material properties produced similar strain distributions to those of orthotropic properties. Again, this is advantageous for studies solely interested in overall strain distributions, and orthotropic material properties are not available or feasible to measure.

In comparison with the experimentally measured average principal tensile strain in the temporal bone, all species-specific isotropic simulations produced lower average strains (figure 8). This resulted in a 45.30 – 52.03% reduction from the experimentally measured average strain (a reduction of 143.52 – 164.84 µε). The finding that the *E*_*Ind* *ML*_ simulation produced higher average strain in comparison with all other isotropic simulations is consistent with observations that the rabbit skull bone properties measured in the ML direction are lower than those in the other orthotropic directions [52]. Interestingly, the four simulations with bone-specific isotropic material properties (e.g. *E*_*Ind* *AP*_) produced strains that were closer to the experimentally measured average strain, resulting in a 45.30 – 49.35% reduction from the experimentally measured average strain (a reduction of 143.52 – 156.36 µε). This is in comparison with average isotropic material properties simulations (e.g. *E*_*All* *AP*_) which produced strains which resulted in a 48.70 – 52.03% reduction from the experimental strain (a reduction of 154.30 – 164.84 µε). These strain differences may only be considered minor, but they suggest that modelling bone-specific material properties produces greater accuracy in predicted strains. Higher strains in the bone-specific isotropic simulations vs average bone isotropic simulations (i.e. *E*_*Ind* *AP*_ vs *E*_*All* *AP*_) was also found in other bones, for example, *E*_*Ind* *AP*_ vs *E*_*All* *AP*_ in the frontal, nasal, premaxilla and alisphenoid bones of Paths 2 and 3 in figure 5. However, it is noted that this was not consistent in every bone and path; for example, the *E*_*All* *AP*_ simulation produced higher strains in the maxilla of Path 1.

Species-specific orthotropic simulations produced much larger region-to-region variations in strain magnitudes when compared with the *E_Datum_* simulation (figures 6–8), and this finding is similar to studies on other species [43–45]. The magnitude of the differences in strain was dependent on the shear modulus modelled, as defining a lower shear modulus (i.e. the ${\bar{E}}_{IndOrth20}$ simulation) produced strains that were noticeably much larger than all other simulations (both isotropic and orthotropic). However, similar strain magnitudes were found between orthotropic simulations with a higher shear modulus (i.e. ${\bar{E}}_{IndOrth5.5}$ vs ${\bar{E}}_{IndOrth4}$ vs ${\bar{E}}_{IndOrth3}$; figure 6). The ${\bar{E}}_{IndOrth20}$ simulation produced the closest comparison with the experimentally measured average strain in the temporal bone (figure 8); while the other orthotropic simulations were closer to the experimentally measured average strain than all isotropic simulations. This would initially suggest that ${\bar{E}}_{IndOrth20}$ is the most accurate simulation; however, figure 6 shows the ${\bar{E}}_{IndOrth20}$ simulation differs from the *E_Datum_* by excess of 2000 µε in regions such as the majority of the zygomatic arch; meaning absolute strain magnitudes exceed 3000 µε. As this study did not experimentally measure strain in the zygomatic arch, we cannot verify the accuracy of the predicted strains in this region. However, such strain magnitudes are suggested to place the region under maximal remodelling, typically occurring to repair damage [78]. Although strains are expected to be high during maximal incisor biting, the use of relatively high muscle forces may not be necessarily uncommon in the rabbit, as incisor biting is commonly used during gnawing to reduce food size. Therefore, we hypothesize that strains of such large magnitude, over vast areas, would suggest the ${\bar{E}}_{IndOrth20}$ simulation overestimates strain magnitude in some bones for a common activity such as maximal biting; thus, orthotropic modelling of skull bone should avoid using a lower shear modulus within the literature, and/or attempt to measure this for the bone under investigation. If the latter is not possible, then the choice of a higher shear modulus may not affect observations of the general strain distributions or strain magnitudes in the skull.

The results of this study are particularly relevant for instances when computational biomechanical modelling of the rabbit is heavily reliant on the validity of simulations such as: application to 3Rs where bone remodelling algorithms can be employed, but outcomes are dictated by the strain magnitudes; or the testing of functional theories that are dependent on strain distribution.

The above observations are made with respect to the limitations of the study, which are common when modelling complex anatomical structures: (i) the observations are limited to homogeneous modelling and are not necessarily transferable to a more accurate representation of variation in material properties through heterogeneous modelling; furthermore, an average elastic modulus per bone was used, and thus did not account for variation in material properties along a direction of measurement within a single bone; (ii) the orthotropic simulations only consider orthotropic properties relating to a global Carteasian coordinate system (AP, ML and VD direction), and properties measured in a cylindrical coordinate system (such as radially, as common when measuring properties in long bones) have the potential to produced altered bone strains to those presented here; (iii) as trabecular bone elastic modulus values was not available for all bone containing trabeculae, the FE model represented both cortical and trabecular bone with the same elastic modulus; however, strains will be influenced by the lower trabecular elastic modulus; (iv) the observations regarding differences between simulations rely on visual inspection of strain distributions; therefore, the significance of such differences is subject to qualitative interpretation; and (v) the elastic modulus used in the orthotropic simulations used the same ratio of shear moduli in each orthotropic direction. Although this is a limitation due to the scarcity of datasets detailing shear moduli in different directions in the same bone, it is likely that shear moduli will vary with respect to the global Cartesian coordinate system used in this study, and may influence the predicted bone strains.

This study has provided an insight into the influence of using material properties measured from mechanical testing upon predicted bone strains in the skull. This is particularly relevant to instances when the material properties of an individual are unknown, and properties from literature are used (which may vary depending on method and location of measurement). This opens the opportunity for future work to compare the predicted bone strains produced from the material modelling methods presented here to those predicted through computed tomography-derived material properties.

In conclusion, the study has highlighted the importance of investigating multiple regions when determining the influence of material properties on strains in the rabbit skull. The results of this study show that modelling orthotropic material properties produces more accurate strain magnitudes, although this is dependent on the shear modulus, with higher moduli producing greater accuracy. In instances when detailed material properties are unknown, it is more accurate to model species-specific elastic modulus (rather than using data from other species), unless the modelling is designed to solely investigate overall distributions. Conclusions are presented with respect to the rabbit, and while these observations may also apply to other lagomorphs and closely related mammals, the extent to which they apply to other species is uncertain and requires further investigation.

## Supplementary Material



## Data Availability

All input data is presented in the paper. Models and scripts are available from the University of Leeds [79]. Supplementary material is available online [80].
