# Supplementary material for: Influence of bone-specific material properties on finite element modelling of the skull
Source: R Soc Open Sci. 2026 Oct 14;13(10):252468. doi: 10.1098/rsos.252468 (PMC13682430; doi:10.1098/rsos.252468)
Supplement: Supplementary file 1 [file rsos.252468.zip › rsos.252468/rsos252468_si_001.pdf]

Electronic Supplementary Material – Bone material properties

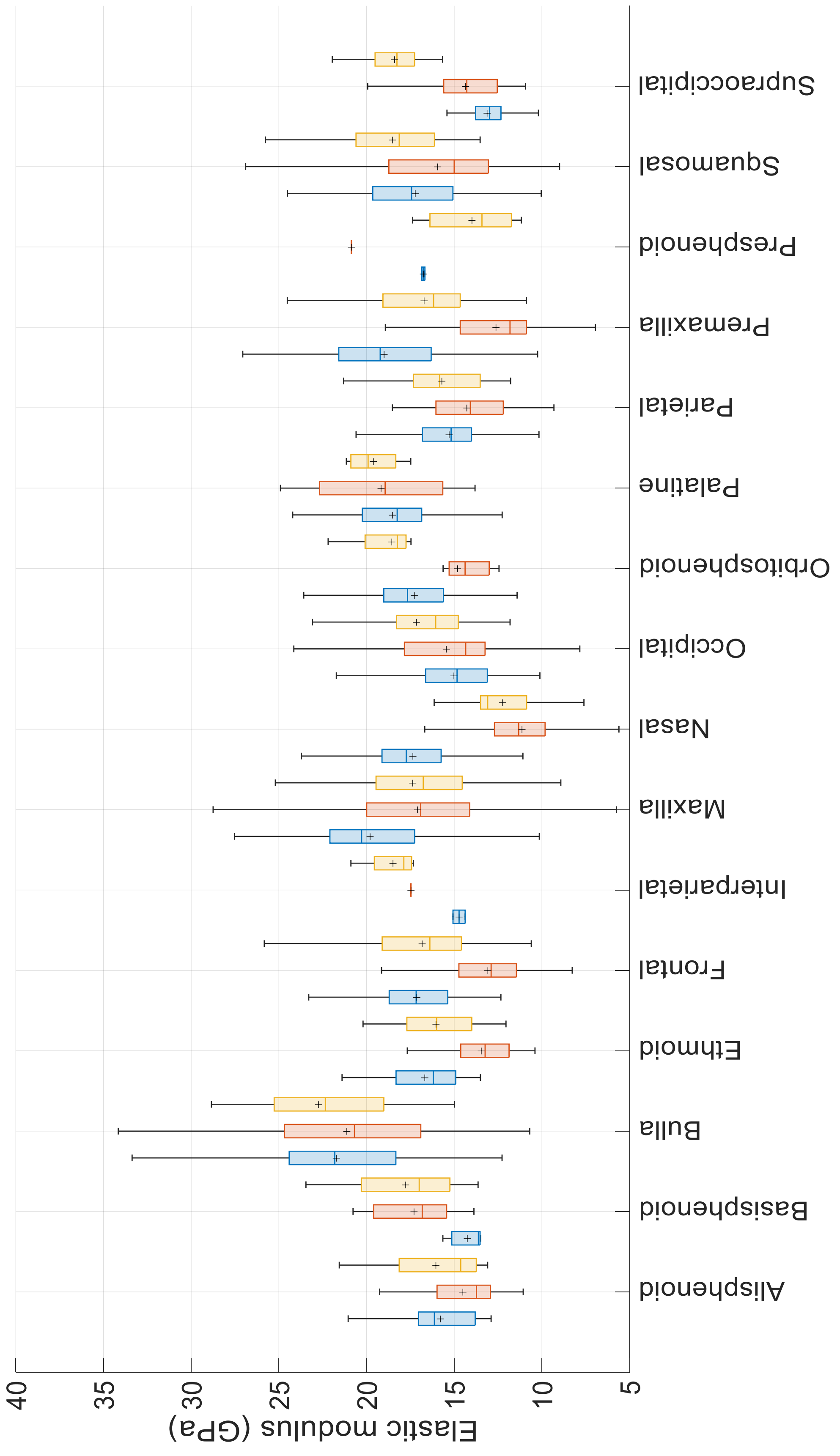

Figure S1. The range of elastic modulus values measured in each bone of the rabbit skull. The box plot illustrates the maximum modulus, minimum modulus, first quartile, third quartile, median and the mean of the modulus values when measured in the anterio-posterior (shown in blue), medio-lateral (shown in red) and ventro-dorsal (shown in yellow) direction. The data represents the elastic modulus values measured in three rabbits. The data was measured in Wang et al (1).

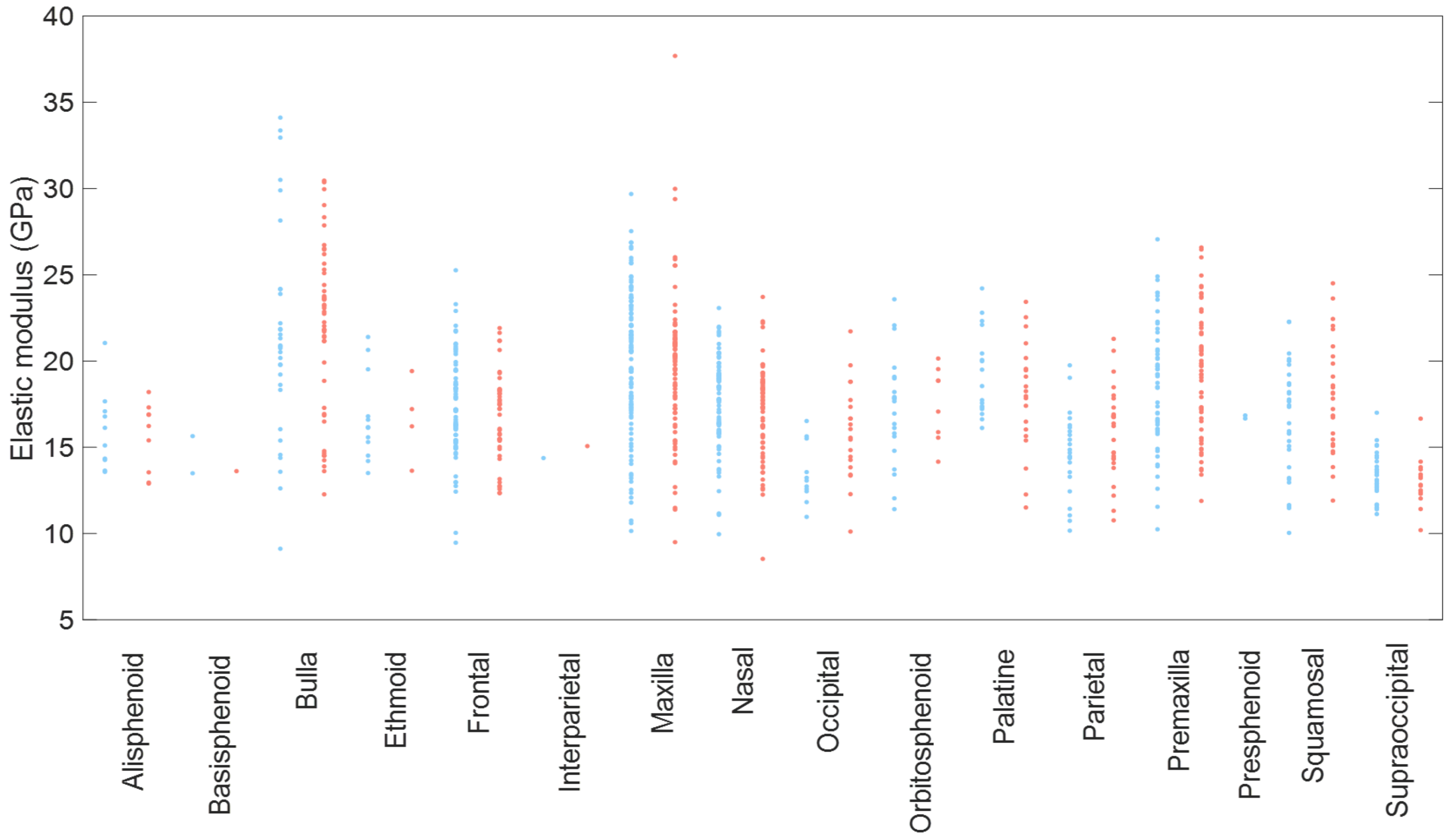

Figure S2. The range of elastic modulus values measured in several skull bones of two separate rabbits. The elastic modulus was measured in the anterio-posterior direction and was measured in Wang et al (1). Blue data points represent rabbit 1; red data points represent rabbit 2.

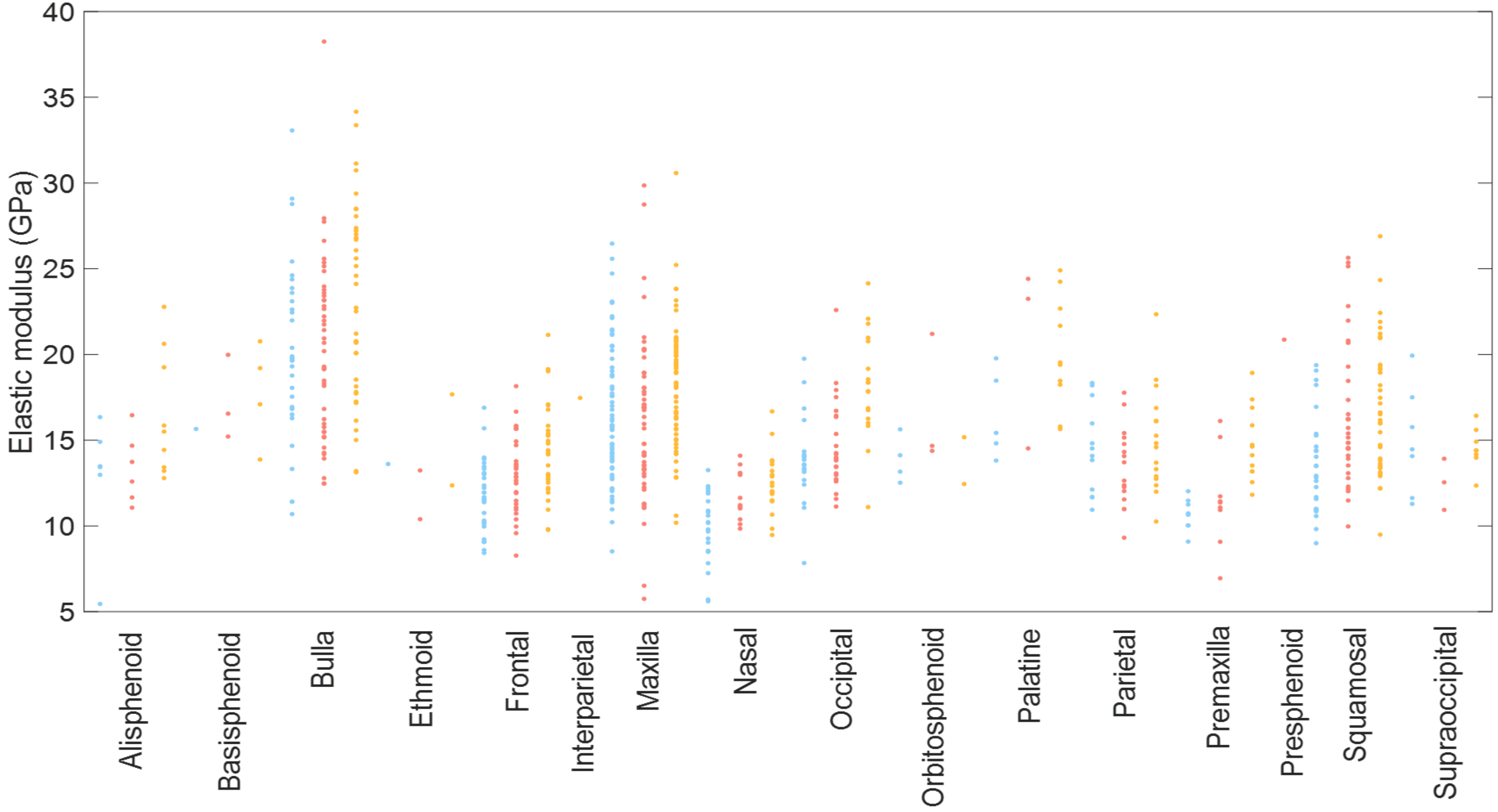

Figure S3. The range of elastic modulus values measured in several skull bones of three separate rabbits. The elastic modulus was measured in the medio-lateral direction and was measured in Wang et al (1). Blue data points represent rabbit 1; red data points represent rabbit 2; yellow data points represent rabbit 3.

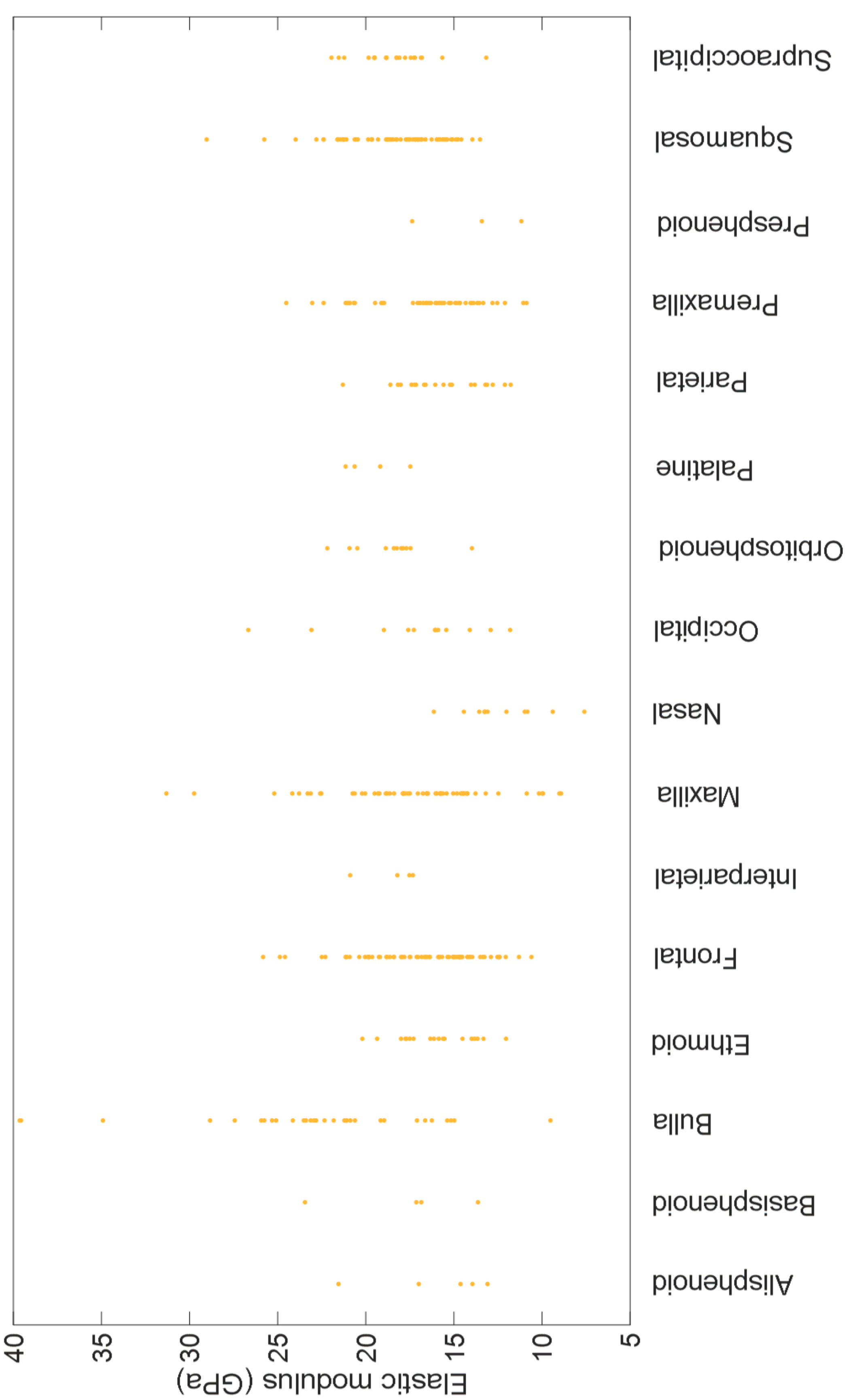

Figure S4. The range of elastic modulus values measured in several skull bones of a single rabbit. The elastic modulus was measured in the ventro-dorsal direction and was measured in Wang et al (1).

1. Wang L, Meloro C, Fagan MJ, Kissane RWP, Bates KT, Askew GN, et al. Regional variation of the cortical and trabecular bone material properties in the rabbit skull. PLoS One. 2024;19: e0298621.
