# Supplementary material for: Influence of bone-specific material properties on finite element modelling of the skull
Source: R Soc Open Sci. 2026 Oct 14;13(10):252468. doi: 10.1098/rsos.252468 (PMC13682430; doi:10.1098/rsos.252468)
Supplement: Supplementary file 1 [file rsos.252468.zip › rsos.252468/rsos252468_si_002.pdf]

Electronic Supplementary Material – Multi-body dynamic modelling

Active muscle force-length curve

An active muscle force-length (*ffl*) curve (Figure S1) was created in MATLAB through fitting a curve to the experimental data of the rabbit digastric muscle reported by Kissane *et al.* (1). The function Smoothing Spline was used to generate a piecewise spline function of normalised muscle force F/F_{om} with respect to normalised muscle fibre length l_{CE}/l_{om}, which is in the range between 0.845 and 1.083. F_{om} is the maximum isometric force. l_{om} is the optimal muscle fibre length and l_{CE} is the muscle fibre length.

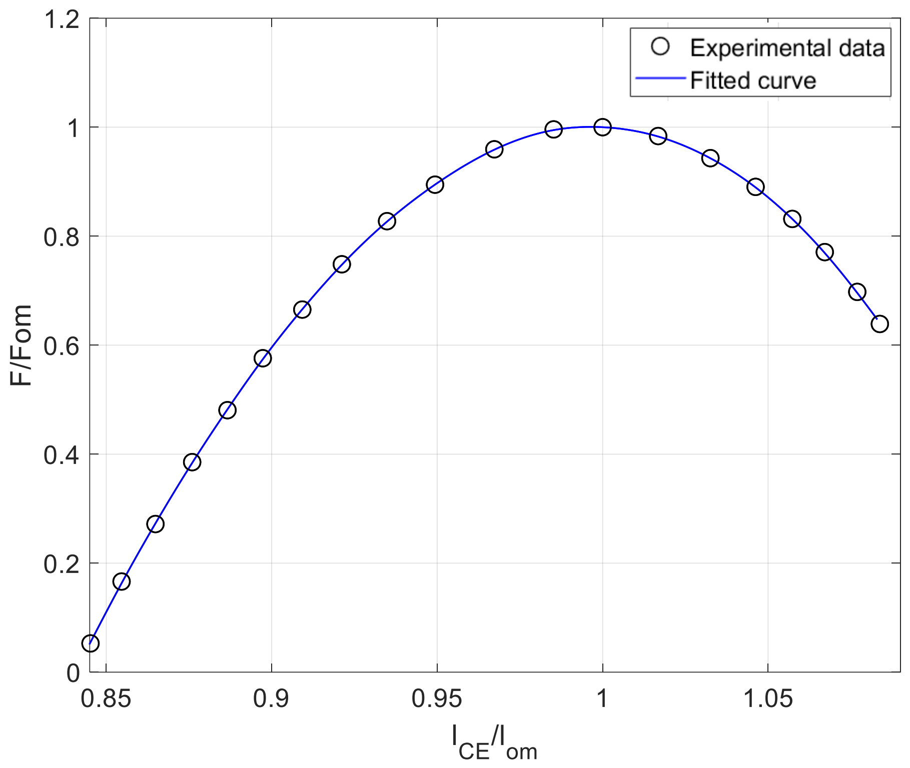

Figure S1. The active muscle force-length (ffl) curve based on the experimental data reported by Kissane *et al.* (1).

Active muscle force-velocity factor curve

A force-velocity (*ffv*) curve (Figure S2) was created in MATLAB through fitting a curve to the experimental data of the rabbit digastric muscle reported by Kissane *et al.* (1). The function Smoothing Spline was used to generate a piecewise spline function of normalised muscle force F/F_{om} with respect to muscle contraction velocity v_{CE}, which is in the range of between -6l_{om} and 10l_{om}.

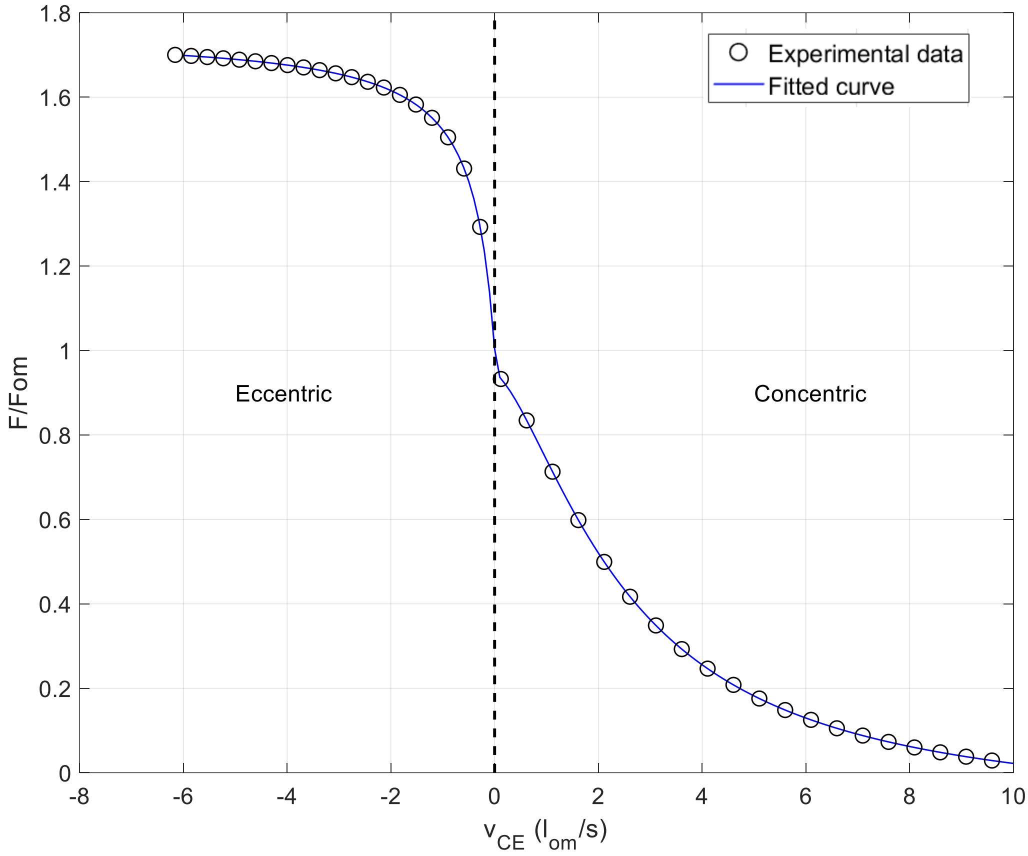

Figure S2. The active muscle force-velocity (ffv) curve based on the experimental data reported by Kissane *et al.* (1).

Passive muscle force-length curve

A passive muscle force-length (ffl_{PE}) curve (Figure S3) was created in MATLAB through fitting a curve to the experimental data of the rabbit digastric muscle reported by Kissane *et al.* (1). The Smoothing Spline function was used to generate a piecewise spline function of normalised muscle force F/F_{om} with respect to normalised muscle fibre length l_{CE}/l_{om}, which is in the range between 1.000 and 1.278.

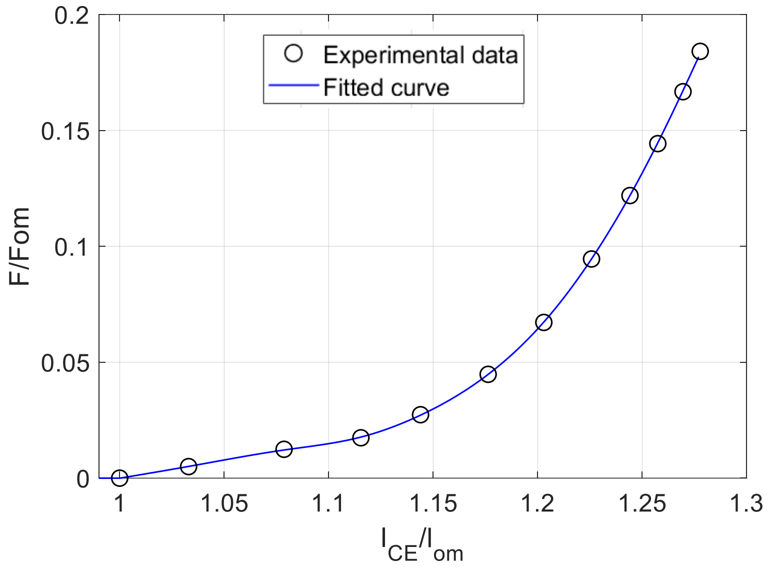

Figure S3. The passive muscle force-length (ffl_{PE}) curve based on the experimental data by Kissane *et al.* (1).

Tendon force-strain curve

Tendon force (F_t) was calculated through Equation 1:

$$F_t = F_{om} \cdot Fl \qquad \textbf{Equation 1}$$

where F_{om} is the maximum isometric force, and Fl is the normalised force relating to the tendon length l_t.

A tendon normalised force-strain (F_l) curve (Figure S4) was calculated using Equation 2 (2, 3):

$$Fl = \begin{cases} 0 & \varepsilon_t \leq 0 \\ 1480.3 \cdot {\varepsilon_t}^2 & 0 \leq \varepsilon_t \leq 0.0127 \\ 37.5 \cdot \varepsilon_t - 0.2375 & \varepsilon_t \geq 0.0127 \end{cases} \qquad \textbf{Equation 2}$$

where $\varepsilon_t = \frac{l_t - l_{ts}}{l_{ts}}$, l_t is the tendon length ($l_{mt} - l_m \cdot \cos\beta$), and l_{ts} is the tendon slack length. The estimation of l_{ts} was based on an optimization algorithm to minimise the differences between three randomly guessed tendon slack lengths (4).

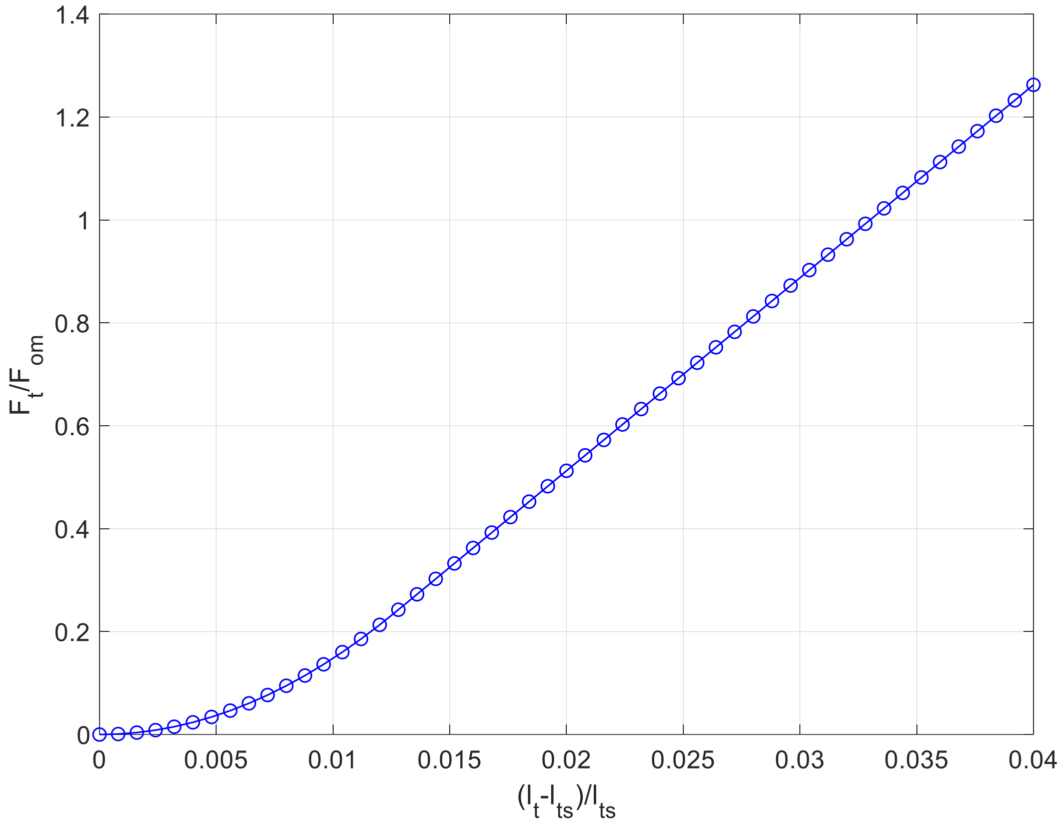

Figure S4. The tendon normalised force strain curve (F_l).

Tendon slack length

As tendon slack length could not be measured directly for each muscle strand, an optimization-based algorithm (4) was used to estimate the tendon slack length l_{ts} for each muscle strand based on minimising tendon slack length changes and applying musculotendon force equilibrium as the optimisation constraint (see the optimisation objective function and constraint equations below). As shown in Figure S5, firstly (Step 1), an input motion trajectory was applied to drive the mandible in ADAMS to acquire a range of musculotendon lengths l_{mt} for each muscle strand. Subsequently (Step 2), three different lengths of l_{mt} were randomly guessed at short (length range between l_{mt_min} and $1.2l_{mt\_min}$), long (length range between $0.8l_{mt\_max}$ and l_{mt_max}) and middle (mean value of the former two) lengths for the first of the 110 muscle strands, and then input into the optimisation constraint equation (Equation 3) to solve the three tendon slack lengths ($l_{ts1}, l_{ts2}, l_{ts3}$), and three muscle fibre lengths (l_{m1}, l_{m2}, l_{m3}). Through specifying 1000 different initial values for the set of three l_m, the optimization calculation ran for 1000 iterations to obtain several solutions for the tendon slack length. These solutions were then averaged to get an estimation for the tendon slack length l_{ts}

in the first guessed set of short, middle and long lengths of l_{mt}. Next (Step 3), the second step was repeated another 9 times by guessing 9 different sets of short, middle and long lengths of l_{mt}. Finally (Step 4), all 10 averaged l_{ts}were averaged to get the final estimation of the tendon slack length for the muscle strand. Steps 2 - 4 were repeated to find the tendon slack length for all other muscle strands.

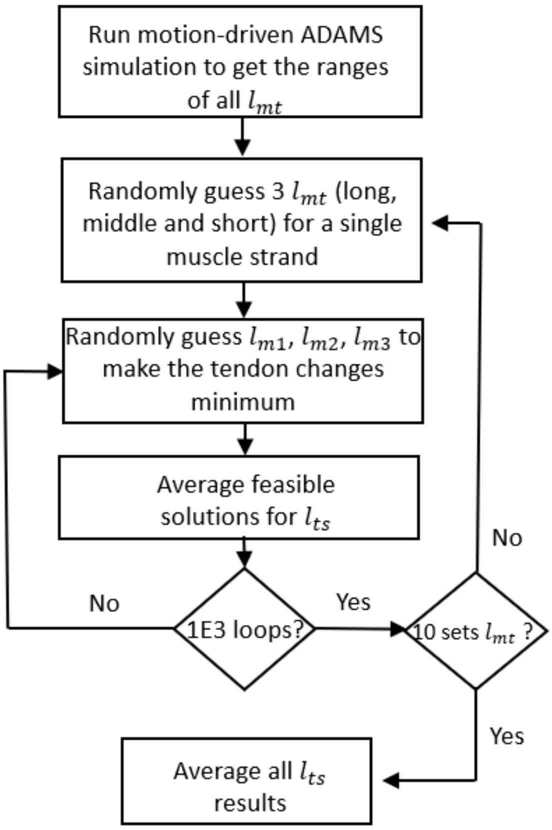

Figure S5. Workflow for the tendon slack length optimisation

Minimising: $$(l_{ts1} - l_{ts2})^2 + (l_{ts1} - l_{ts3})^2 + (l_{ts3} - l_{ts2})^2$$

Constraint:

$$\begin{cases} \left[aF_{max}ffl(l_{m1})ffv(\dot{l}_m) + F_{om}ffl_{PE}(l_{m1})\right]\cos\beta = F_{om}ffl_t(l_{mt1} - l_{m1}\cos\alpha - l_{ts1})/l_{ts1} \\ \left[aF_{max}ffl(l_{m2})ffv(\dot{l}_m) + F_{om}ffl_{PE}(l_{m2})\right]\cos\beta = F_{om}ffl_t(l_{m2} - l_{m2}\cos\alpha - l_{ts2})/l_{ts2} \\ \left[aF_{max}ffl(l_{m3})ffv(\dot{l}_m) + F_{om}ffl_{PE}(l_{m3})\right]\cos\beta = F_{om}ffl_t(l_{mt3} - l_{m3}\cos\alpha - l_{ts3})/l_{ts3} \end{cases}$$

Equation 3

where a is the muscle activation and assumed to be 1; F_{max} is the maximum muscle force; $ffl(l_m)$ is the active muscle force-length curve function with regard to the variable, muscle fibre length l_m; $ffv(\dot{l}_m)$ is the muscle force-velocity curve function; F_{om} is the optimal isometric muscle force that

is equal to maximum muscle force; $ffl_{PE}(l_m)$ is the passive muscle force-length curve function; β is the pennation angle; ffl_t is the tendon force-length curve function and l_{ts} is the tendon slack length.

References

1. Kissane RWP, Bates KT, Fagan MJ, Wang L, Watson PJ, Askew GN. The functional role of the rabbit digastric muscle during mastication. *J Exp Biol*. 2024;227(18):jeb249238.

2 Kuo P-H, Deshpande AD. Contribution of passive properties of muscle-tendon units to the metacarpophalangeal joint torque of the index finger. 3rd IEEE RAS & EMBS International Conference on Biomedical Robotics and Biomechatronics, Tokyo, Japan, 2010: 288-294.

3. Zajac F. Muscle and tendon: properties, models, scaling, and application to biomechanics and motor control. I. 1989;17(4):359-411.

4. Manal K, Buchanan TS. Subject-specific estimates of tendon slack length: A numerical method. *J. Appl. Biomech*. 2004;20(2):195–203.
